# In Situ and Real‐Time Multi‐Modality Imaging Guided Orderly Triple‐Therapy of Tumors with a Multifunctional Nanodrug

**DOI:** 10.1002/advs.202501048

**Published:** 2025-04-24

**Authors:** Chaoyi Yang, Yuexuan Meng, Ying An, Jing Jia, Yuru Wang, Guangming Li, Yiran Li, Shan Wu, Chengyao Geng, Yunlong Chen, Huangxian Ju

**Affiliations:** ^1^ State Key Laboratory of Analytical Chemistry for Life Science School of Chemistry and Chemical Engineering Nanjing University Nanjing 210023 P. R. China

**Keywords:** multi‐modality imaging, nanodrug, triple‐therapy, tumor

## Abstract

Effective integration of different therapeutic methods is a promising way to improve the overall efficacy of tumor therapy, which needs to be guided by in situ and real‐time monitoring of each therapeutic process. Here a multifunctional AuNR@SiO_2_@MnO_2_@DNA prodrugs (ASMD) nanodrug is designed for orderly photothermal therapy (PTT)/chemodynamic therapy (CDT)/gene therapy (GT) triple‐therapy of tumors, which can be guided by the in situ and real‐time photoacoustic (PA)/magnetic resonance (MR)/fluorescence (FL) multi‐modality imaging. The gold nanorod in ASMD can generate a PA signal and perform PTT. The MnO_2_ in ASMD can respond to the glutathione inside tumor cells to release Mn^2+^, which can generate MR signal and perform CDT by catalyzing the degradation of intracellular H_2_O_2_ to generate **·**OH. The DNA prodrugs can perform a cascade response in the presence of the released Mn^2+^ and the intracellular microRNA 21, which can turn on the quenched FL signal and release small‐interfering RNA and antisense oligonucleotide to perform GT. Guiding by the in situ and real‐time PA/MR/FL multi‐modality imaging of each therapeutic process, an orderly PTT/CDT/GT triple‐therapy of tumors is established, which provides a significant and promising strategy to develop more efficient and practical therapeutic programs for tumors.

## Graphical abstract

A multifunctional AuNR@SiO_2_@MnO_2_@ DNA prodrugs (ASMD) nanodrug is designed to establish an in situ and real‐time photoacoustic (PA)/magnetic resonance (MR)/fluorescence (FL) multi‐modality imaging guided orderly photothermal therapy (PTT)/chemodynamic therapy (CDT)/gene therapy (GT) triple‐therapy of tumor.

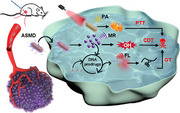

## 1 Introduction

Tumors are still one of the deadliest diseases of humans.^[^ ^1^ ^]^ So far, various methods have been developed for the therapy of tumors, which mainly include photothermal therapy (PTT),^[^ ^2^ ^]^ photodynamic therapy (PDT),^[^ ^3^ ^]^ chemodynamic therapy (CDT),^[^ ^4^ ^]^ gene therapy (GT)^[^ ^5^ ^]^ and immunotherapy.^[^ ^6^ ^]^ Nevertheless, each method suffers inherent defects. For example, PTT and PDT have efficient short‐term curative effect but are not significant for long‐term and deep tumors.^[^ ^7^ ^]^ CDT exhibits a longer‐term curative effect, but its efficiency is often limited by the antioxidant systems in tumors.^[^ ^8^ ^]^ GT has the advantages of long‐term effects and individualized treatment, but still surfers potential risks of systemic toxicity caused by prodrug leakage and invalidity caused by oligonucleotide degradation.^[^ ^9^ ^]^ Immunotherapy is a revolutionary method compared to traditional anti‐tumor therapy, but still has complexity and uncertainty including the severe adverse reactions caused by over‐active immune systems.^[^ ^10^ ^]^ Some nanoparticles/nanofiber‐related therapy for tumors have also been developed,^[^ ^11^, ^12^, ^13^, ^14^ ^]^ but single method‐based therapeutic strategies are often insufficient throughout the entire course of the tumor treatment. Integration of different methods is an ingenious solution,^[^ ^15^ ^]^ which can remedy the defects of each method to improve the overall efficacy of tumor therapy.

However, the simple combination of different methods sometimes cannot improve the overall efficacy, which may even lead to lower efficacy or worse feedback due to the mutual interference of each method. An effective integration must orderly initiate different therapeutic processes under an optimal order, which need the guidance of the in situ and real‐time process feedback. To achieve this goal, the in situ and real‐time monitoring of the therapeutic process of each method is a critical issue.^[^ ^16^ ^]^ Multi‐modality imaging is a technology that combines different imaging techniques, including fluorescence (FL),^[^ ^17^ ^]^ magnetic resonance (MR),^[^ ^18^ ^]^ photothermal (PT), photoacoustic (PA),^[^ ^19^ ^]^ ultrasound (US),^[^ ^20^ ^]^ X‐ray computed tomography (CT),^[^ ^21^ ^]^ positron emission tomography (PET)^[^ ^22^ ^]^ and so on. It can overcome the limitations of each independent imaging modality and obtain higher‐quality and more valuable information,^[^ ^23^ ^]^ which is the ideal technology to in situ and real‐time monitor the therapeutic process of different therapeutic methods.

**Scheme 1:**
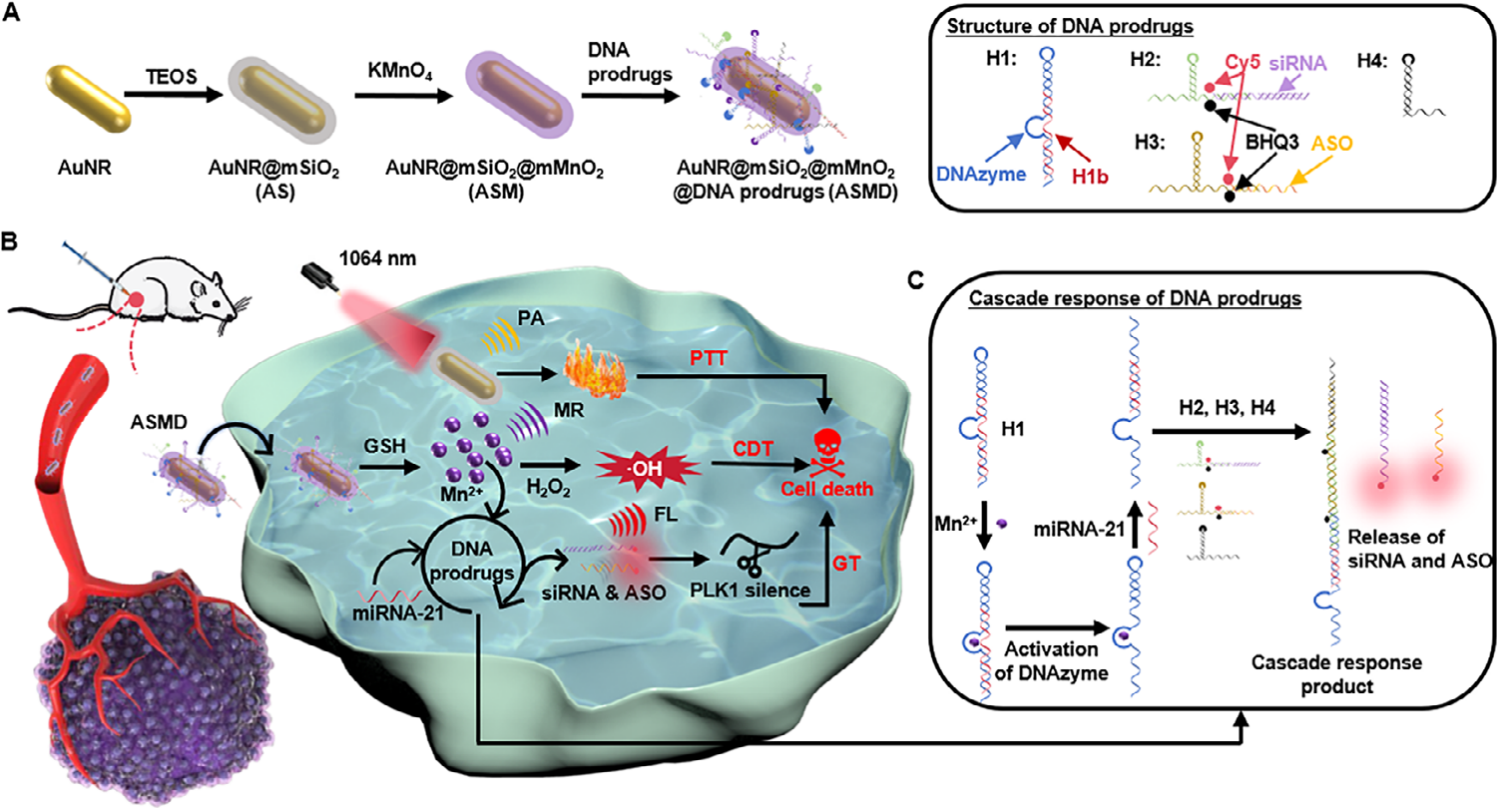
Schematic illustrations. A) Preparation of ASMD nanodrugs. B) PTT/CDT/GT triple‐synergistically precise therapy of tumors guided by in situ and real‐time PA/MR/FL multi‐modality imaging. C) Mn^2+^ and miRNA‐21 synergistically initiated cascade response of DNA prodrugs.

Herein this work, a multifunctional AuNR@SiO_2_@MnO_2_@ DNA prodrugs (ASMD) nanodrug was designed, which can achieve an orderly PTT/CDT/GT triple‐therapy of tumors guided by in situ and real‐time PA/MR/FL multi‐modality imaging. The ASMD was constructed using a gold nanorod (AuNR) absorbing in a near‐infrared (NIR) II window^[^ ^24^ ^]^ as the substrate (Scheme 1A), which was first coated with a layer of silica oxide (SiO_2_) on the surface by tetraethoxysilane (TEOS) hydrolysis reaction.^[^ ^25^ ^]^ The obtained AuNR@SiO_2_ (AS) was further coated with a layer of manganese dioxide (MnO_2_) to obtain AuNR@SiO_2_@MnO_2_ (ASM) through potassium permanganate (KMnO_4_) decomposition^[^ ^26^ ^]^ on the unreacted organosilica.^[^ ^24b^ ^]^ Finally, the ASM was loaded with a set of DNA prodrugs through electrostatic adsorption^[^ ^27^ ^]^ to obtain the ASMD, which contained four different DNA hairpins (H1–H4) (Scheme 1A; Table S1, Supporting Information). H1 contained a hairpin tail and a Mn^2+^ responsive DNAzyme sequence (H1a),^[^ ^27^ ^]^ which was blocked with a complementary sequence (H1b) to prevent the unfolding of the hairpin by the target RNA. H2 and H3 were two hairpins (H2a, H3a), which were respectively tethered with Cy5 labeled small‐interfering RNA (siRNA) and antisense oligonucleotide (ASO)^[^ ^28^ ^]^ both targeting tumor‐associated gene polo‐like kinase 1 (PLK1)^[^ ^27^ ^]^ as gene drugs through the corresponding complementary overhang. PLK1 is a key regulator of cellular proliferation that is over‐expressed in many malignant cells, which is also an oncogenic target for gene therapy.^[^ ^29^ ^]^ The synergistic treatment of siRNA and ASO can effectively improve the efficacy of gene therapy.^[^ ^30^ ^]^ The two Cy5 dyes were both quenched by BHQ3 that modified on the corresponding site on H2 and H3. H4 was the auxiliary sequence for the cascade hybridization. After being delivered to the tumor region and endocytosed by tumor cells (Scheme 1B), the overexpressed glutathione (GSH)^[^ ^27^ ^]^ first degraded the MnO_2_ shell to produce Mn^2+^ and release DNA prodrugs. Under an exaction of a 1064 nm laser, the AuNR could generate photothermal and PA signals for PTT and PA imaging.^[^ ^24^ ^]^ The produced Mn^2+^ can generate **·**OH by catalyzing the degradation of intracellular H_2_O_2_ and MR signal for CDT and MR imaging.^[^ ^31^ ^]^ Besides, the Mn^2+^ could further act as the co‐ions of DNAzyme in H1 to cut the corresponding nucleic acid site of H1b and expose a 10‐mer toehold for the recognition of tumor‐specific microRNA 21^[^ ^32^ ^]^ (miRNA‐21) (Scheme 1C). After hybridized with miRNA‐21 to unfold the hairpin in H1, the cascade hybridizations from H1 to H4 were initiated, which could sequentially release the dangled siRNA and ASO to efficiently silence the PLK1 gene for GT and recover the Cy5 fluorescence for FL imaging. According to the in situ and real‐time PA/MR/FL multi‐modality imaging results, the different processes of PTT/CDT/GT were successfully monitored, which guided the establishment of an orderly PTT/CDT/GT therapeutic program. As a proof of concept, the lung cancer cell A549^[^ ^33^ ^]^ tumor‐bearing mice are used as models to demonstrate the designed multi‐modality imaging guided orderly multi‐therapy strategy, which provides a significant and promising tool for clinical tumor therapy.

## 2 Results and Discussion

### 2.1 Preparation and Characterization of ASMD Nanodrug

The AuNRs were prepared according to the previously reported procedure^[^ ^24^ ^]^ and first characterized by transmission electron microscopy (TEM) and UV–vis–NIR spectrometry. As revealed in TEM image (Figure S1, Supporting Information), the miniature AuNRs had the highest long‐aspect‐ratios of ≈6.0 (e.g., length of ≈42.5 nm; width of ≈7.0 nm). After sequentially coating with SiO_2_ and MnO_2_, the obvious layers of SiO_2_ and MnO_2_ were observed in the TEM images of AS and ASM with good monodispersities (Figure S2, Supporting Information). Meanwhile, the absorption peaks of AuNRs exhibited a 10 nm redshift from 1064 to 1074 nm after coated with SiO_2_, which further shifted to 1114 nm and exhibited an extra absorption peak at 383 nm after coated with MnO_2_ (Figure S3, Supporting Information), demonstrating the successful coating of SiO_2_ and MnO_2_ layers on ASs and ASMs. The successful construction of the DNA prodrugs was demonstrated by gel electrophoresis analysis (Figure S4, Supporting Information). To avoid the interference of fluorescent dye and quencher in gel imaging, nucleotides without fluorescence and quencher modification (H2a‘, siRNA‘, H3a‘, ASO‘) were used in in vitro validation (Table S1, Supporting Information). Individual bands of H1, H2′ and H3′ was observed in the corresponding lane (Figure S4, Supporting Information, Lanes 4, 7, 10), which demonstrated the successful hybridization of H1b to H1a and tethering of siRNA′ and ASO′ to H2a′ and H3a′. After being loaded with DNA prodrugs, an extra nucleic acid characteristic 260 nm absorption peak^[^ ^34^ ^]^ appeared (Figure S3, Supporting Information). Besides, obvious Si, Mn, O, P, and N elements were observed around the Au elements in the element mapping images (Figure S5A, Supporting Information) and energy‐dispersive X‐ray spectroscopy (EDS) spectra (Figure S5B, Supporting Information) of ASMD, which further demonstrated the successful coating of SiO_2_ and MnO_2_ and loading of the DNA prodrugs on ASMD. The loading rate of DNA prodrugs onto ASM was investigated by UV–vis spectrometry and gel electrophoresis analysis of the remaining contents of DNA prodrugs after loading onto ASM (Figure S6, Supporting Information), which was calculated to be 98% with an ASM to DNA prodrugs mass ratio of 4:1, indicating the economic efficiency of the designed nanodrug.

The step‐by‐step modification of AS, ASM, and ASMD was further verified by dynamic light scattering (DLS), zeta potential, X‐ray photoelectron spectroscopy (XPS) survey spectrum analysis, and Fourier‐transform infrared spectroscopy (FTIR). The DLS and the zeta potential analysis exhibited a obviously increased hydrated diameters and gradually negatively charged potentials of AuNR, AS, ASM, and ASMD (Figures S7 and S8, Supporting Information). The XPS spectrum of ASM exhibited the correct composition of Au, Mn, and O elements (Figure S9A, Supporting Information). The characteristic peaks at 652.75 and 641.25 eV corresponding to the Mn 2p3/2 and Mn 2p1/2 spin–orbit peaks of MnO_2_^[^ ^35^ ^]^ suggested the +4 valence state of Mn element in ASM (Figure S9B, Supporting Information), which demonstrated the existence of MnO_2_ in the nanodrugs. The FTIR spectra exhibited characteristic absorbance peaks of Si─O─Si and Mn─O bonds at 1093 and 532 cm^−1^ in AS and ASM, respectively (Figure S10, Supporting Information), which further demonstrated the successful step‐by‐step modification.

### 2.2 In Vitro Functions of ASMD Nanodrug

**Figure 1:**
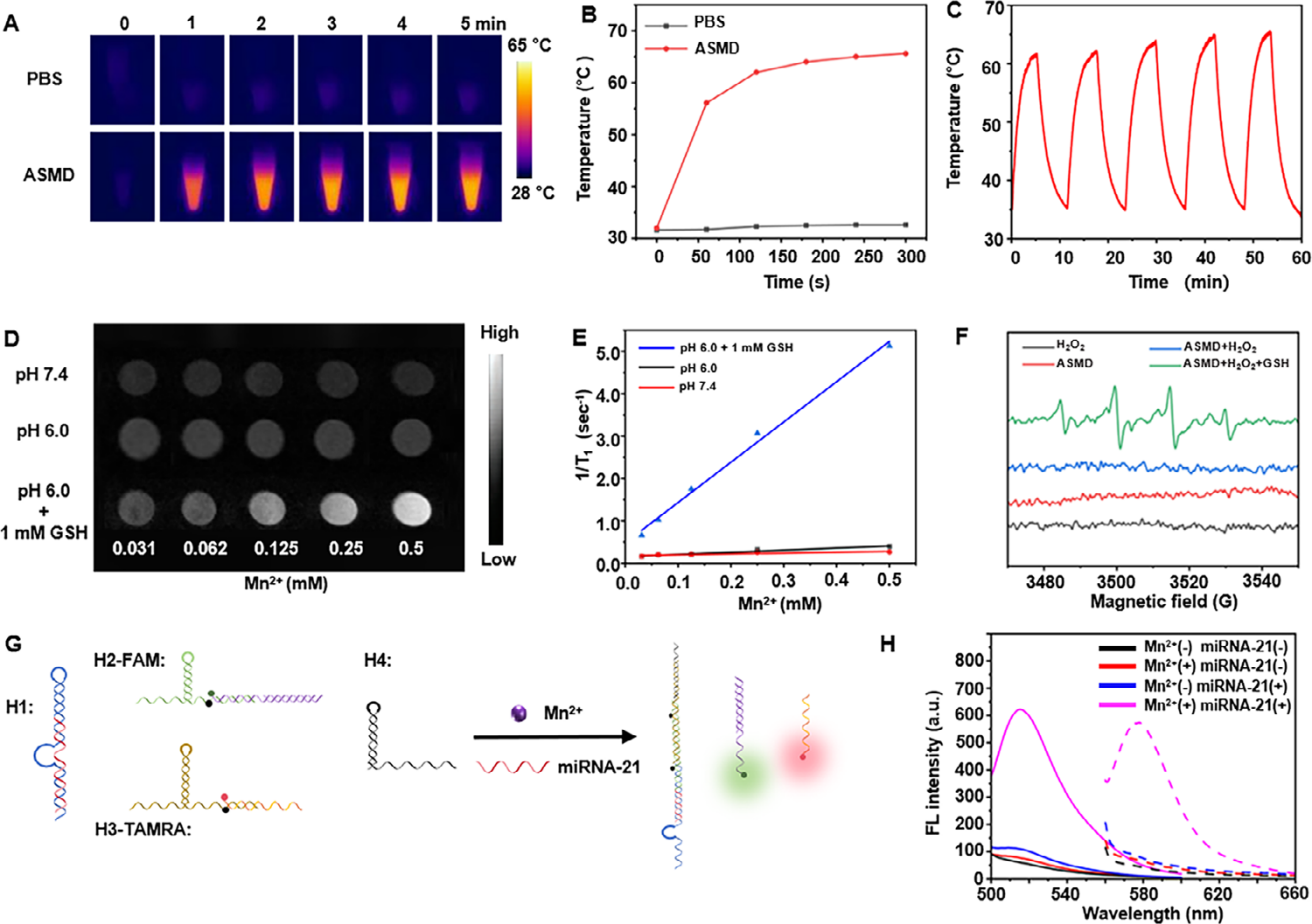
In vitro photothermal effect, MR performance, cascade response of DNA prodrugs of ASMD nanodrug. A) Infrared thermal images of PBS and 400 µg mL^−1^ of ASMD solution upon 1064 nm laser irradiation at a power density of 0.8 W cm^−2^ from 1 to 5 min. B) Temperature curves of PBS and ASMD solution from (A). C) Temperature curves of 400 µg mL^−1^ of ASMD solution upon five on/off cycles of 1064 nm laser irradiation at a power density of 0.8 W cm^−2^. D) T1‐weighted MR images of different Mn^2+^ concentrations of ASMD solutions under different pH or with extra GSH (1 mm). The Mn^2+^ concentrations of ASMD solutions were measured by ICP‐AES analysis. E) Fitting curves of the 1/T1 intensities to Mn^2+^ concentrations from (D). F) ESR spectra of ASMD solutions under different conditions. (Buffer: pH 6.0 PBS containing 25 mm NaHCO_3_) G) Illustration of Mn^2+^ and miRNA‐21 synergistically initiated cascade response of DNA prodrugs to release siRNA and ASO. H) FAM (solid lines) and TAMRA (dashed lines) fluorescence spectra of the mixture of four DNA prodrugs treated with Mn^2+^ and miRNA‐21, respectively.

The photothermal effect of ASMD nanodrug was first investigated by in vitro infrared thermal imaging (**Figure** **1**A). 400 µg mL^−1^ of AuNR, As, ASM, and ASMD solutions were irradiated by a 1064 nm laser, and the real‐time temperature was recorded by the near‐infrared camera, respectively. After 5 min of laser irradiation (Figure 1B; Figure S11, Supporting Information), the temperature of all the solutions exhibited similar temperature variations, indicating the negligible influence of the photothermal effects after the addition of the shell multilayer substance to the Au nanorods. The temperature could rise to 65.9 °C and exhibited a stable photothermal effect after five lasers on/off cycles (Figure 1C), demonstrating the excellent photothermal property of ASMD nanodrug for PA imaging and PTT.

As the magnetic moment and paramagnetic center of Mn^2+^ could enhance the T_1_ MR signal and lead to a large longitudinal relaxation rate,^[^ ^35^ ^]^ the GSH‐activated degradation of MnO_2_ on ASMD was investigated by in vitro T_1_ MR imaging and longitudinal relaxivity analysis (Figure 1D,E). After incubating different concentrations of ASMD solutions with 1 mm GSH in pH 6.0 PBS buffer for 20 min, obvious color change of the ASMD solutions was observed (Figure S12, Supporting Information). After subjecting these solutions to MR analysis, an enhanced r_1_ signal up to 9.504 mm ^−1^ s^−1^ was detected, while these incubated without GSH only exhibited 0.494 and 0.179 mm ^−1^ s^−1^ in pH 6.0 and 7.4, respectively (Figure 1E). The rates of the released Mn^2+^ under different conditions were quantified by inductively coupled plasma‐atomic emission spectrometry (ICP‐AES) (Figure S13, Supporting Information), which exhibited 93% in pH 6.0 containing 1 mm GSH, but only 25% and 10% in pH 6.0 and 7.4. Besides, the TEM images also exhibited the disappeared shell structure on ASMD only after being treated with pH 6.0 PBS containing 1 mm GSH (Figure S14, Supporting Information). These results demonstrated the successful release of Mn^2+^ from ASMD by GSH, which could be used as the tumor cell‐activated T_1_ contrast agent for MR imaging.

Next, the Mn^2+^ triggered Fenton‐like chemical reaction of H_2_O_2_ to generate **·**OH^[^ ^31^ ^]^ was verified by electron spin resonance (ESR) spectroscopy using 5, 5‐dimethyl‐1‐pyrroline‐N‐oxide (DMPO), which could identify **·**OH. After incubating ASMD with 1 mm of GSH and 100 µM of H_2_O_2_ in the PBS buffer (pH 6.0 containing 25 mM NaHCO_3_) and then subjected to ESR analysis, obvious **·**OH signals were observed (Figure 1F), while these without GSH exhibited negligible signal of **·**OH, demonstrating the feasibility by utilizing Mn^2+^ to generate **·**OH for CDT.

Finally, the cascade response of DNA prodrugs (Figure 1G) was investigated in vitro. The released contents of DNA prodrugs from ASMD under different conditions were quantified by UV–vis spectrometry (Figure S15, Supporting Information), which exhibited 85% in pH 6.0 containing 1 mm GSH, but only 17% and 7% in pH 6.0 and 7.4. The activation of DNAzyme in H1 by Mn^2+^ was investigated by incubating different concentrations of Mn^2+^ with H1 at 37 °C for 2 h. After subjecting to gel electrophoresis analysis (Figure S16, Supporting Information), an obvious division of H1 band was observed when incubating with 0.5 mm of Mn^2+^, demonstrating the successful activation of DNAzyme in H1. The Mn^2+^ and miRNA‐21 synergistically initiated cascade response of DNA prodrugs were investigated by incubating different combinations of DNA hairpins with miRNA‐21 and Mn^2+^ at 37 °C for 2 h (Figure S17, Supporting Information). The mixture of four DNA hairpins (H1, H2′, H3′, H4) did not exhibit any interactions when individually incubated with miRNA‐21 and Mn^2+^ (Figure S17, Supporting Information, Lanes 2, 4, 5), but exhibited obvious band of cascade response production and the released siRNA’ and ASO when simultaneously incubated with miRNA‐21 and Mn^2+^ (Figure S17, Supporting Information, Lane 6). To distinguish the release of siRNA and ASO, H2‐FAM and H3‐TAMRA were used in fluorescence spectrometry analysis by replacing the Cy5/BHQ3 pairs in H2 and H3 to FAM/BHQ1 and TAMRA/BHQ2, respectively (Table S1, Supporting Information). The recovery of both FAM and TAMRA fluorescence was observed only when the mixture of four DNA prodrugs treated with both Mn^2+^ and miRNA‐21 (Figure 1H, and S18, Supporting Information), which further demonstrated the Mn^2+^ and miRNA‐21 synergistically initiated cascade response of DNA prodrugs to release siRNA and ASO for GT and FL imaging.

### 2.3 In Vitro PTT/CDT/GT Mediated Cell‐Killing of ASMD Nanodrug

The cellular uptake efficiency of ASMD was first evaluated by incubating A549 cells with a Cy5‐labeled DNA (Table S1, Supporting Information) loaded ASM (ASM‐Cy5) and subjected to confocal laser scanning microscopy (CLSM) imaging, which exhibited an efficient internalization of ASM‐Cy5 by A549 cells after 4 h (Figure S19, Supporting Information). To investigate the cellular uptake pathways of ASM‐Cy5, the A549 cells were pre‐treated including chlorpromazine hydrochloride (CPZ, clathrin‐mediated endocytosis inhibitor),^[^ ^36^ ^]^ Nystatin (Nys, caveolin‐mediated endocytosis inhibitor),^[^ ^37^ ^]^ and Ethylisopropylamiloride (EIPA, macropinocytosis inhibitor).^[^ ^28^ ^]^ After being incubated with ASM‐Cy5 and subjected to CLSM imaging, only the Nys‐treated cells exhibited obvious internalization of ASM‐Cy5 (Figure S20, Supporting Information), which indicated the clathrin‐mediated endocytosis and micropinocytosis of ASM‐Cy5.

To investigate the Mn^2+^ triggered CDT cell‐killing of cells, the cell viabilities after internalized with different concentrations of AuNR, AS or ASM were investigated by CCK8 assay (Figure S21, Supporting Information). After incubation for 4 or 24 h, only the ASM‐treated cells exhibited an obvious decrease in cell viability with concentrations of 50 and 100 µg mL^−1^. To demonstrate the Mn^2+^ triggered Fenton‐like chemical reaction of H_2_O_2_ to generate **·**OH in living cells, the reactive oxygen species (ROS) staining dye 2′,7′‐dichlorofluorescin diacetate (DCFH‐DA)^[^ ^31^ ^]^ was used to detect the oxidative stress of cells treated with different concentrations of ASM. After being subjected to CLSM imaging (Figure S22, Supporting Information), obvious fluorescence of DCFH was observed on the cells incubated with increased concentrations of ASM, which demonstrated the feasibility of the Mn^2+^‐triggered CDT cell‐killing of cells.

**Figure 2:**
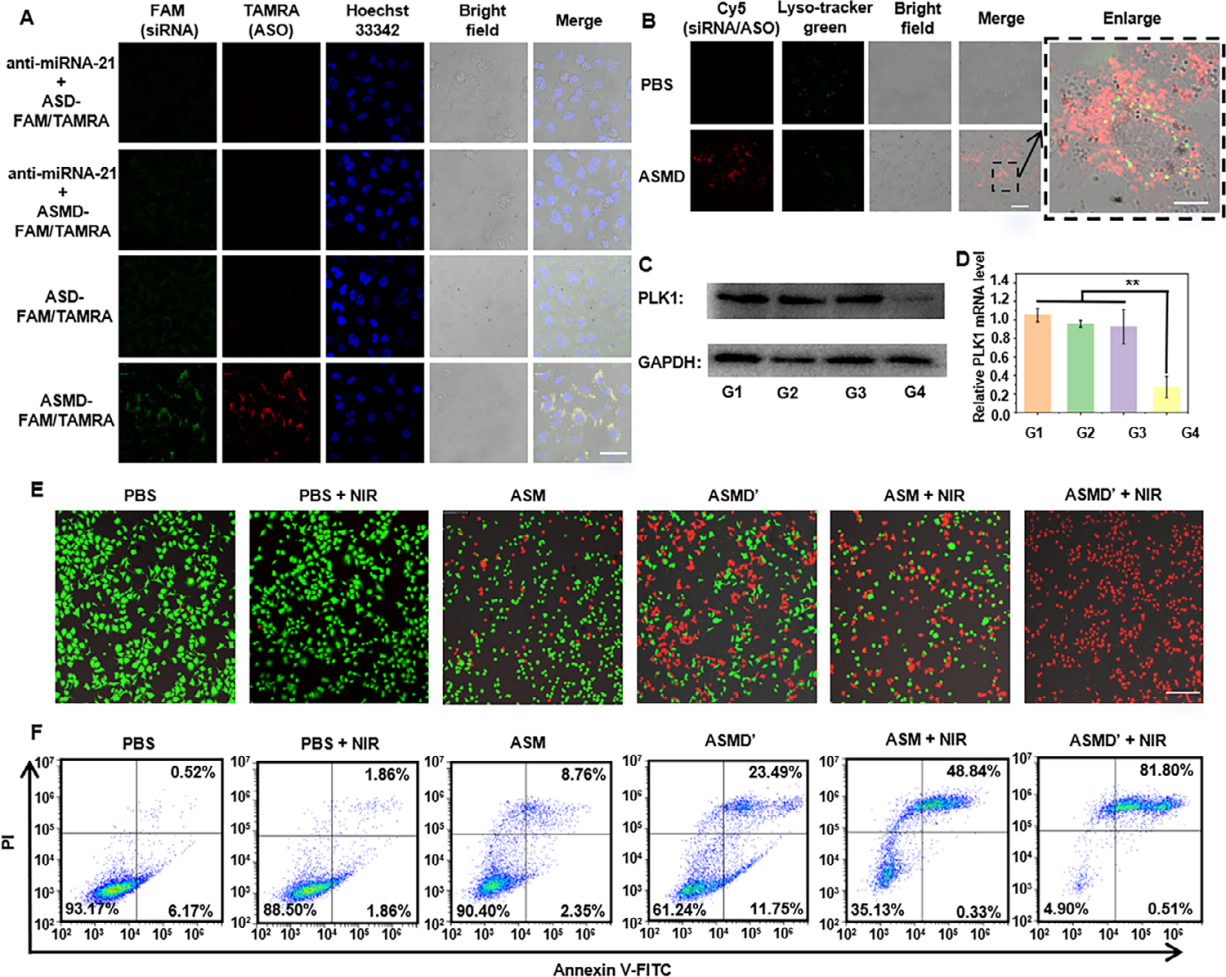
Release of DNA prodrugs inside cells and in vitro PTT/CDT/GT mediated cell‐killing of ASMD. A) CLSM images of A549 cells or anti‐miRNA‐21 pre‐treated A549 cells incubated with ASD‐FAM/TAMRA or ASMD‐FAM/TAMRA before staining with Hoechst. Scale bar: 20 µm. B) CLSM images of A549 cells incubated with ASMD and then stained with Lyso‐track green. Scale bar: 40 µm. Scale bar in enlarged image: 10 µm. C,D) Western blotting (C) and qPCR (D) analysis of PLK1 proteins and RNAs extracted from A549 cells (G1), A549 cells treated with anti‐miRNA‐21 and then ASMD (G2), A549 cells treated with ASD (G3), A549 cells treated with ASMD (G4). The data were analyzed using a Student's *t*‐test at a significance level of *p* > 0.05 (NS), *p* < 0.05 (^*^), *p* < 0.01 (^**^), *p* < 0.001 (^***^). (E, F) CLSM images E) and flow cytometry analysis F) of A549 cells treated with PBS, ASM, and ASMD’ with or without NIR irradiation, respectively. Scale bar: 200 µm.

The Mn^2+^ and intracellular miRNA‐21 synergistically triggered release of DNA prodrugs was first investigated by using an Mn^2+^‐free nanodrug (ASD) and the anti‐miRNA‐21 pre‐treated cells to block miRNA‐21.^[^ ^38^ ^]^ To distinguish the release of siRNA and ASO, ASD‐FAM/TAMARA and ASMD‐FAM/TAMRA were prepared by using H2‐FAM and H3‐TAMRA instead of H2 and H3 (Table S1, Supporting Information), respectively. The A549 cells and anti‐miRNA‐21 pre‐treated A549 cells were treated with ASD‐FAM/TAMARA and ASMD‐FAM/TAMRA at 37 °C for 6 h, respectively. After subjecting to CLSM imaging, only the cells treated with ASMD‐FAM/TAMRA exhibited obvious FAM and TAMRA fluorescence (**Figure** **2**A), which indicated the Mn^2+^ and intracellular miRNA‐21 synergistically triggered release of siRNA and ASO. To investigate the possible lysosomal trapping of the released siRNA and ASO from ASMD, the cells treated with ASMD were further treated cells with Lyso‐tracker green dye for 1 h. After subjecting to CLSM imaging, no obvious overlap between the Lyso‐tracker green and Cy5 fluoresce was observed (Figure 2B), which indicated the negligible impact of lysosomal trapping of DNA prodrugs.

Since the efficient delivery and release of DNA prodrugs inside cells has been confirmed, the functions of siRNA and ASO were further investigated by western blotting and qPCR assay of the PLK1 expression in the treated cells. The A549 cells were respectively treated with anti‐miRNA‐21 then ASMD (G2), ASD (G3), and ASMD (G4) at 37 °C for 6 h. Cells without any treatment were set as the control group (G1). After the standard cell lysis, and protein/RNA extraction procedure, the protein and RNA samples from each group were subjected to western blotting and qPCR assay. As a result, G4 exhibited the lowest expression of PLK1 proteins (Figure 2C) and mRNA level (Figure 2D), while G2 and G3 did not exhibit significant differences compared to G1, demonstrating the Mn^2+^ and miRNA‐21 synergistically triggered inhibition of the PLK1 protein by the DNA nanodrugs for GT.

Finally, the in vitro PTT/CDT/GT mediated cell‐killing by ASMD nanodrug was investigated by propidium iodide (PI)/calcine AM living/dead cell staining^[^ ^39^ ^]^ (Figure 2E) and CCK8 assay (Figure S23, Supporting Information). To avoid the interference of fluorescent dye and quencher in living/dead cell staining, the H2 and H3 in ASMD were replaced with H2′ and H3′ without fluorescence and quencher modification to obtain ASMD‘ (Table S1, Supporting Information). A549 cells were respectively treated with ASM, and ASMD′ at 37 °C for 6 h with or without 1064 nm NIR irradiation, which could be corresponded to different combinations of PTT, CDT, and GT. After staining with PI/calcine AM for CLSM imaging (Figure 2E) or Annexin V‐FITC/PI for flow cytometry analysis (Figure 2F), the ASM‐treated cells (CDT) exhibited tiny red fluorescence with an 8.76% of dead cells, while these combined with NIR irradiation (PTT/CDT) and the ASMD′ treated cells (CDT/GT) exhibited a larger area of red fluorescence (Figure 2E) and 48.84% and 23.49% of the dead cells (Figure 2F). In contrast, the cells treated with ASMD’ and simultaneously irradiated by NIR irradiation (PTT/CDT/GT) all exhibited red fluorescence (Figure 2E) and 81.80% of the dead cells (Figure 2F), which indicated the most efficient killing of the cells. Similar flow cytometry results were observed on HeLa and MCF‐7 cells (Figure S24, Supporting Information). These results were consistent with the CCK8 assay of all three cell lines (Figures S23 and S25, Supporting Information), which demonstrated the great in vitro cell‐killing efficiency of the PTT/CDT/GT triple‐therapy with ASMD nanodrugs.

### 2.4 In Vivo PA/MR/FL Multi‐Modality Imaging Guided Orderly PTT/CDT/GT Triple‐Therapy of Tumors with ASMD Nanodrug

**Figure 3:**
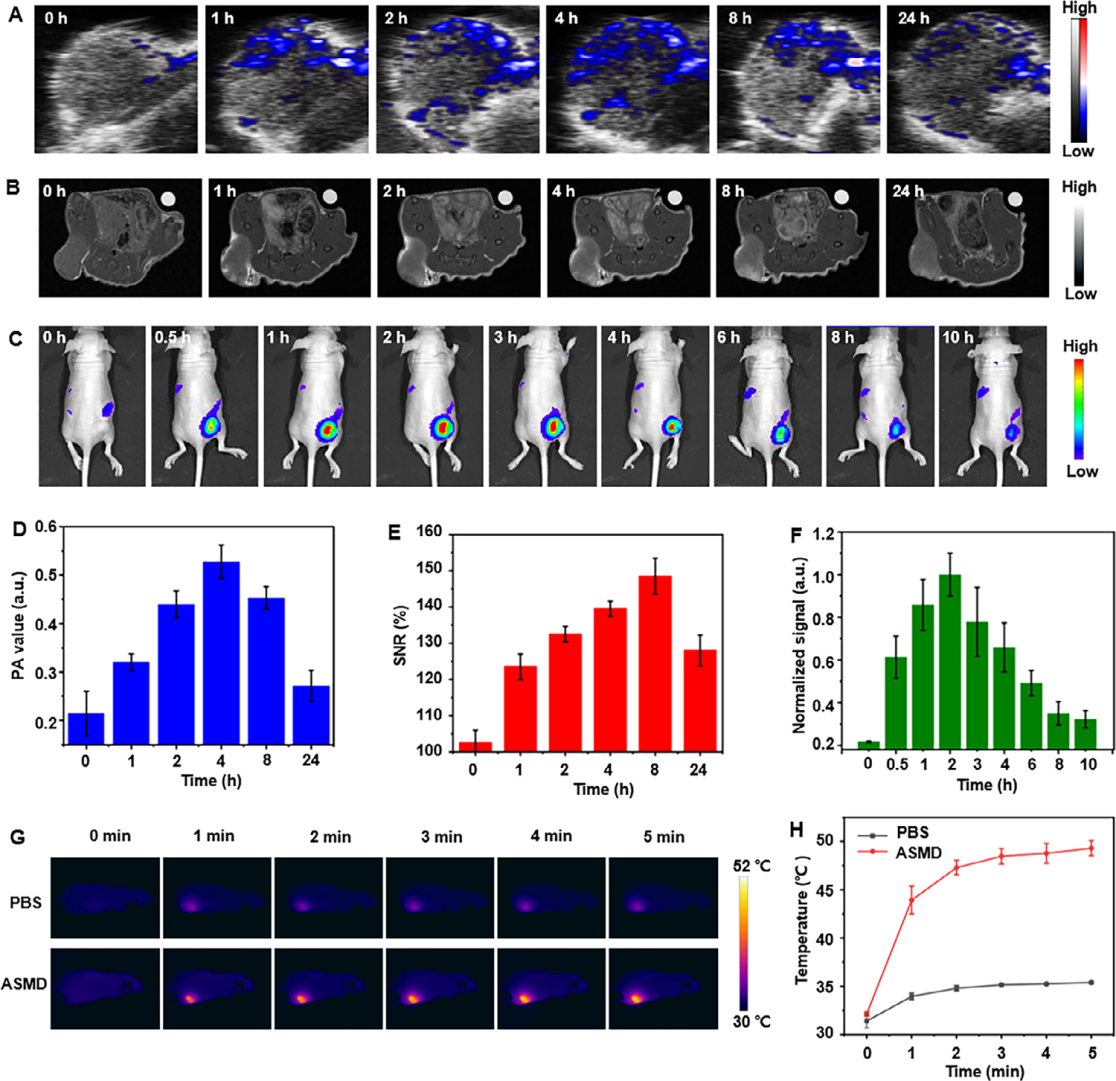
In vivo PA/MR/FL multi‐modality imaging of A549 tumor‐bearing mice. A–C) In vivo PA (A), MR (B) and FL (C) images of A549 tumor‐bearing mice after injected with ASMD for different time. B) In vivo GSH‐activated MRI of ASMD NPs in A549 tumor‐bearing mice. D–F) The corresponding PA values at 808 nm (D), signal to noise ratio (SNR) of MRI signals (E) normalized the FL signal of the tumor region at different times from (A)–(C). G) In vivo thermal images of the tumor regions on A549 tumor‐bearing mice after injected with PBS or ASMD and irradiated under 1064 nm laser (0.8 W cm^−2^, 5 min). H) Temperature variation curves from (G).

To demonstrate the in vivo PA/MR/FL multi‐modality imaging capability of ASMD nanodrugs, the A549 subcutaneous tumor xenograft mice were peritumorally injected with ASMD nanodrug (dose: 4 mg kg^−1^) and subjected to PA, MR and FL imaging (**Figure** **3**A–C). A weak PA signal at the tumor region was observed 1 h after injection, which gradually enhanced and reached a maximum PA value 4 h after injection (Figure 3D). Meanwhile, the MR signal in the tumor region was obviously observed 1 h after injection, which also gradually enhanced and reached a maximum SNR value 8 h after injection (Figure 3E). Besides, the FL signal has appeared in the tumor region 0.5 h after injection, which reached a maximum FL value of 2 h after injection and then gradually decreased within another 2 h (Figure 3F). These phenomena indicated that the ASMD could concentrate into the tumor region within 4 h after injection, which could be efficiently internalized and continuously release Mn^2+^ through GSH degradation inside tumor cells within 8 h after injection. Moreover, the DNA prodrugs loaded on ASMD could be maximumly released 2 h after injection, which could be basically completed within another 2 h before performing the gene silence process. To achieve the highest PTT efficacy and reduce the impact of PTT on the gene silence process, the 1064 nm laser (0.8 W cm^−2^) was irradiated on the tumor region 4 h after injection for 5 min. Compared to the mouse without nanodrug injection, the mouse injected with ASMD exhibited an obvious temperature increase up to 52 °C on the tumor region (Figure 3G,H), which indicated the feasibility of PTT and the temperature could be easily withstood by the DNA prodrugs (Figure S26, Supporting Information). Thus, the in vivo multi‐modality imaging capability of the designed strategy could provide important guidance for the order of different therapeutic processes to achieve the maximization of efficacy.

**Figure 4:**
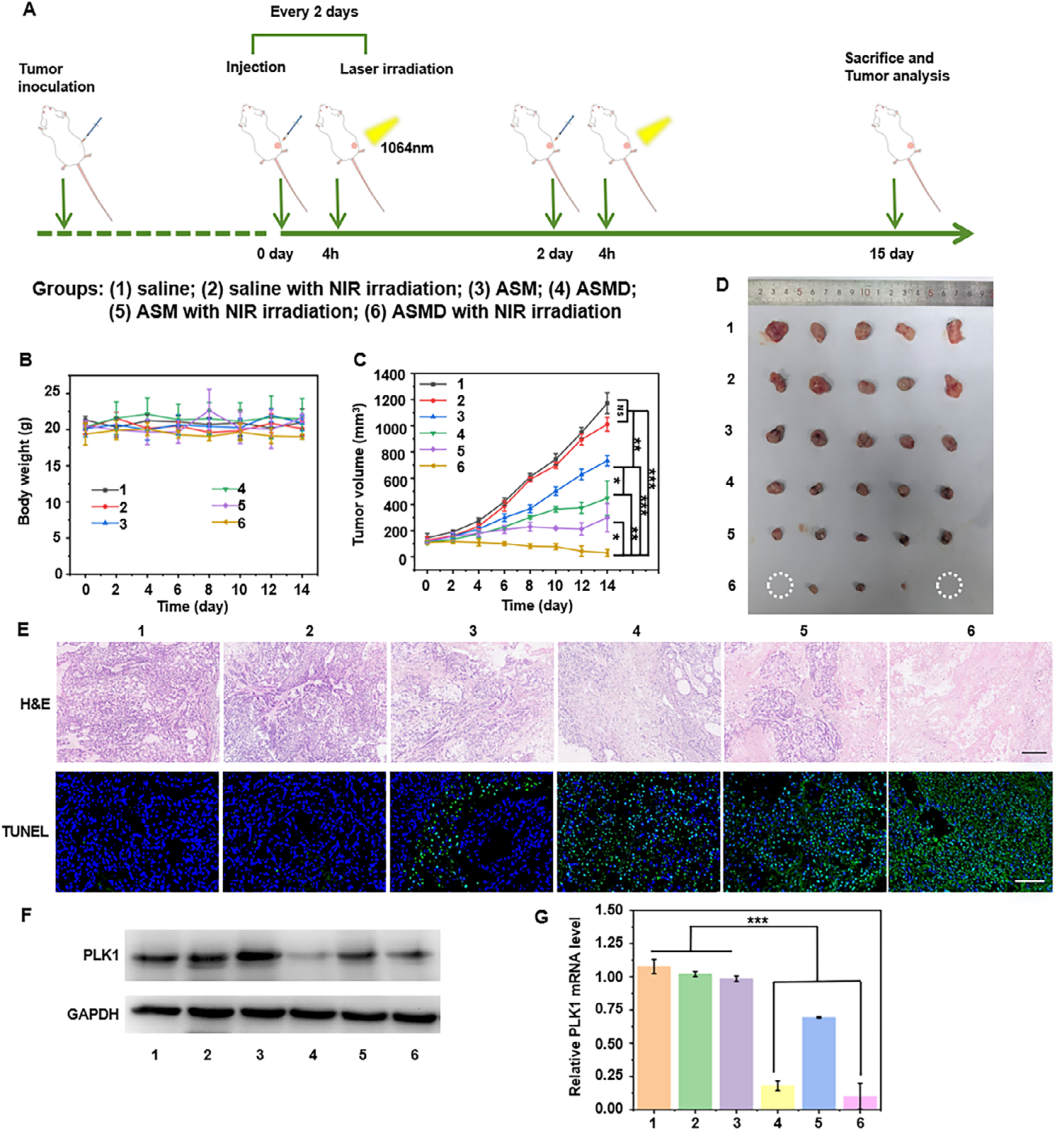
Orderly PTT/CDT/GT triple‐therapy of A549 tumor‐bearing mice. A) Schematic illustration for the treatment of A549 tumor‐bearing mice with ASMD nanodrugs and 1064 nm laser irradiation. The mice were divided into 6 groups: 1) saline; 2) saline with NIR irradiation; 3) ASM; 4) ASMD; 5) ASM with NIR irradiation; 6) ASMD with NIR irradiation. B,C) Variation of body weight (B) and tumor volume (C) of different groups of A549 tumor‐bearing mice. D) Photographs of dissected tumors from different groups of mice at day 15. E) Histology and CLSM images of sectioned tumor tissues from different groups of mice after H&E and TUNEL staining. Scale bar in H&E staining: 200 µm; Scale bar in TUNEL staining: 100 µm. F,G) Western blotting (F) and qPCR (G) analysis of PLK1 proteins and RNAs extracted from tumors from different groups of mice. The data were analyzed using a Student's *t*‐test at a significance level of *p* > 0.05 (NS), *p* < 0.05 (^*^), *p* < 0.01 (^**^), *p* < 0.001 (^***^).

The efficacy of the designed orderly PTT/CDT/GT triple‐therapy of tumors was investigated by using six groups of A549 tumor‐bearing mice (5 mice per group). After the tumor volume rose to ≈100 mm^3^, different groups of mice were respectively treated with 1) saline, 2) saline with NIR irradiation, 3) ASM, 4) ASMD, 5) ASM with NIR irradiation, 6) ASMD with NIR irradiation by peritumoral injection and 1064 nm laser (0.8 W cm^−2^) irradiation at tumor region every other day for 14 days, respectively (**Figure** **4**A). According to the PA/MR/FL multi‐modality imaging results (Figure 3A–F), the NIR irradiation was performed 4 h after injection and lasted 10 min. The in vivo blood half‐life (t_1/2_) and metabolic circulation of ASMD after peritumoral injection was measured to be 6.3 and 48 h by inductively coupled plasma‐mass spectrometry (ICP‐MS) detection of the contents of Au in the blood (Figure S27, Supporting Information) and major organs (Figure S28, Supporting Information). There was no discernible difference in the body weight of all mice (Figure 4B; Figure S29, Supporting Information), and no pathological abnormality of their heart, liver, spleen, lung, and kidney in the pathological observation (Figure S30, Supporting Information), and negligible hemolysis rate within 48 h in the hemolysis test (Figure S31, Supporting Information), which demonstrated the negligible side effects and biosafety during these treatments. The mice of groups 1 and 2 exhibited tiny variations of the tumor volumes (Figure 4C,D; Figure S32, Supporting Information), indicating no therapeutic effect by only using NIR irradiation. Meanwhile, the mice of groups 3, 4, and 5 exhibited a gradually enhanced inhibition of the tumor volume (Figure 4C,D; Figure S32, Supporting Information) with inhibition rates of 38.9%, 59%, 77% (Figure S33, Supporting Information), which could be respectively attributed to the ASM‐induced CDT, ASMD‐induced CDT, and GT, and ASM with NIR irradiation‐induced PTT and CDT. Moreover, the mice of group 6 exhibited maximum inhibition and even complete ablation of the tumors (Figure 4C,D; Figure S32, Supporting Information) with an inhibition rate of 98% (Figure S33, Supporting Information). These results indicated the greatest efficacy of the ASMD with NIR irradiation‐induced PTT, CDT, and GT of tumors. The pathological states of tumor tissues dissected from each group were assessed by hematoxylin and eosin (H&E) and terminal deoxynucleotidyl transferase dUTP nick end labeling (TUNEL) assay (Figure 4E).

The H&E and TUNEL staining images from the mice of group 6 displayed the largest necrotic area and the highest level of cell apoptosis, which was in good agreement with the variations in tumor volumes. Thus, the designed orderly PTT/CDT/GT triple‐therapy could exactly achieve optimal efficacy of tumors from the aspects of these three therapeutic methods.

To further validate the orderly PTT/CDT/GT triple‐therapy of tumors by ASMD nanodrug, the PLK1 protein and mRNA levels in the tumor tissues sectioned from the mice of 6 different groups were analyzed by WB and qPCR assay (Figure 4F,G). As a result, the tumor tissues from groups 1–3 and 5 exhibit no significant difference or only a slight decrease of both PLK1 protein (Figure 4F) and mRNA levels (Figure 4G), while those from groups 4 and 6 exhibited a similarly lowest expression of both PLK1 protein (Figure 4F) and mRNA levels (Figure 4G). These results further demonstrated the GT efficacy induced by the DNA prodrugs on ASMD, and the efficient reduction of the interference between different therapeutic processes guided by the multi‐modality imaging.

## 3 Conclusion

In conclusion, this work establishes an in situ and real‐time PA/MR/FL multi‐modality imaging guided orderly PTT/CDT/GT triple‐therapy of tumors by using an ASMD nanodrug, which achieves a great in vitro cell‐killing efficiency of tumor cells and an optimal efficacy of tumors in vivo. According to the in vivo PA/MR/FL multi‐modality imaging result, the time‐sequenced initiation of PTT, CDT, and GT was ordered by the control of NIR irradiation, which achieves the maximization of the efficacy of tumor therapy. The ASMD can be conveniently prepared and exhibits good biosafety. The PA and MR properties of ASMD provide the application potentials of ASMD in deeper tumors. The DNA prodrugs loaded on ASMD can be simply changed to other GT targets to expand the applications of various tumors. By introducing more therapeutic methods, the proposed multi‐modality imaging guided orderly multiple‐therapy provides a significant and promising strategy to develop more efficient and practical therapeutic programs for tumors.

## Conflict of Interest

The authors declare no conflict of interest.

## Supporting information



Supporting Information

## Data Availability

The data that support the findings of this study are available from the corresponding author upon reasonable request.
