# Supplementary material for: In Situ and Real‐Time Multi‐Modality Imaging Guided Orderly Triple‐Therapy of Tumors with a Multifunctional Nanodrug
Source: Adv Sci (Weinh). 2025 Apr 24;12(25):2501048. doi: 10.1002/advs.202501048 (PMC12224957; doi:10.1002/advs.202501048)
Supplement: Supplementary file 1 — Supporting Information [file ADVS-12-2501048-s001.pdf]

ADVANCED SCIENCE
Open Access

Supporting Information

for *Adv. Sci.*, DOI 10.1002/advs.202501048

In Situ and Real-Time Multi-Modality Imaging Guided Orderly Triple-Therapy of Tumors with a Multifunctional Nanodrug

Chaoyi Yang, Yuexuan Meng, Ying An, Jing Jia, Yuru Wang, Guangming Li, Yiran Li, Shan Wu, Chengyao Geng, Yunlong Chen and Huangxian Ju**

Experimental section

Materials and reagents

All chemicals and solvents used were in analytic level. Millipore water was used to prepare all aqueous solutions. All oligonucleotides were synthesized and characterized by Sangon Biotech (China). The unmodified DNA strands were purified by polyacrylamide gel electrophoresis. The modified DNA strands were purified by High-Performance Liquid Chromatography. A549 cell lines were purchased from the China Infrastructure of Cell Line Resource. $HAuCl_4 \cdot 4H_2O$ (≥ 99.9%), TEOS (≥ 99%), Hexadecyl trimethylammonium bromide (CTAB, ≥ 99%), $AgNO_3$ (99.8%), sodium borohydride ($NaBH_4$, 98%) and hydroquinone were purchased from Sigma-Aldrich Chemistry Co., Ltd. $KMnO_4$ (99.5%) was obtained from Nanjing Chemical Reagent Co., Ltd. Hoechst 33342, RIPA lysis buffer and PVDF membranes were purchased from Thermo Fisher Scientific. Cell Counting Kit-8 (CCK-8), 4% paraformaldehyde solution, BSA-PBST, and TBST were purchased from Sangon Biotech Co., Ltd (Shanghai). Anti-glyceraldehyde-3-phosphate dehydrogenase (GAPDH) antibody and anti-Polo-like Kinase 1 (PLK1) antibody were purchased from Beyotime Biotechnology (Shanghai, China). Ham's F-12K, phosphate buffered saline (PBS), and trypsin and fetal bovine serum (FBS) were obtained from Biochannel Technology Co., Ltd (Nanjing). Trypsin, 2′,7′-dichlorofluorescein diacetate (DCFH-DA), calcein AM, Lyso-Tracker Green and Annexin V-FITC/PI cell apoptosis kits were obtained from KeyGen Biotech. Co. Ltd (Nanjing). HRP-conjugated Goat anti-mouse IgG (secondary antibody) and super enhanced chemiluminescence (ECL) detection reagent were purchased from FUDE Technology Co., Ltd (Hangzhou). FastPure Complex Cell/Tissue Total RNA Isolation kit were acquired from Vazyme Biotech Co. Ltd (Nanjing, China). PrimeScript™ RT reagent Kit and TB Green® Premix Ex Taq™ II (Tli RNaseH Plus) were obtained from Takara Bio (Japan). Deionized (DI) water (18.4 MΩ) used in all experiments was produced by Millipore Elix 5 water purification system.

Apparatus

Dynamic light scattering (DLS) analysis was conducted on a BI-200SM wide angle dynamic and static laser light scatterer (Brookhaven, USA). Zeta potentials were measured on a Nano-Z Zetasizer (Malvern, UK). UV-vis-NIR absorption spectra were recorded with a UV-3600 spectrophotometer (Shimadzu, Japan). The high-resolution transmission electron microscopic (TEM) images were collected on a JEM-2800 high-resolution transmission electron microscope (JEOL, Japan). Element mapping images and Energy-dispersive X-ray spectroscopy (EDS) spectrum were collected via a Tecnai G2 F20 High resolution transmission electron microscope (HRTEM) (FEI, USA). The measurement of DNA prodrug was carried out on a NanoDrop One/One^C microvolume UV-Vis Spectrophotometer (Thermo Fisher Scientific, USA). Fourier transform infrared spectra (FT-IR) were recorded with Nicolet iS50 FT-IR spectrometer (Thermo Fisher Scientific, USA). The gel electrophoresis was acquired on a PowerPac™ basic electrophoresis analyzer (Bio-Rad, USA). Fluorescence spectra were recorded on an F-7100

fluorescence spectrophotometer (Hitachi, Japan). CCK-8 assay was performed on a Multiskan FC microplate reader (Thermo Fisher Scientific, USA). Confocal laser scanning microscope (CLSM) images were acquired with a Leica TCS SP8 confocal laser scanning microscope (Leica, Germany) and a Novel NCF950 confocal laser scanning microscope (Novel, China). Flow cytometric analysis was conducted on Cytoflex S flow cytometer (Beckman Coulter, USA). The quantitative real-time PCR (qRT-PCR) testing was carried out on StepOnePlus™ real-time fluorescence PCR system (Applied Biosystems, USA). The imaging of the mice was conducted on an IVIS Lumina XR III in vivo imaging system (PerkinElmer, USA). In vitro measurement of Mn element was performed with an Avio500 inductively coupled plasma atomic emission spectrometer (ICP-AES) (PE, USA). In vivo measurement of Au element was performed with an Agilent 7850 inductively coupled plasma-mass spectrometer (ICP-MS) (Agilent, USA). MR relaxivity was measured on a 0.5 T MR scanner (NMI20-015 V-I, NIUMAG). All MR imaging experiments were performed on a 1.0 T small animal MR scanner (Bruker ICON™). All photoacoustic (PA) imaging experiments were accomplished with a Vevo LAZR-X imaging system (FUJIFILM VisualSonics, Canada). Photothermal images were recorded by an infrared thermal (IR) imaging instrument (Fotric 225-1, China).

1. Preparation of nanodrugs

The AuNR used in this work was prepared according to previous report.[24] In a typical procedure, 1.6 mL of $HAuCl_4$ (aq) (10 mM) and 90 μL of $AgNO_3$ (aq) (0.1 M) were added to 40 mL of CTAB (aq) (0.1 M). Then, 100 μL of HCl (1 M) and 2.1 mL of aqueous hydroquinone (0.1 M) were added to the growth solution under gentle mixing. The color of the growth solution turned from orange to clear with light yellow color. After 15 min of stirring, 160 μL of freshly prepared ice cold $NaBH_4$ (aq) (10 mM) solution was injected into the growth solution using micro-syringe. The mixture was stirred vigorously for 15 s and aged for 16 h at 37 °C. The obtained AuNR solution was then purified by centrifuging at 12000 rpm for 15 min twice.

To prepare AuNR@SiO_2 (AS), the AuNR solution was re-dispersed in 40 mL of ultrapure water and adjusted to pH 9-10 by ammonia (28 wt.%).[21,26] Then 20 μL of TEOS (10 wt.% in ethanol) was added into the above solution at 30 min intervals under gentle stirring for three times. The mixture was continually stirred for 24 h at 37 °C. The obtained AS was washed by centrifugation twice (10000 rpm for 10 min) and re-dispersed with 20 mL of ultrapure water.

To prepare AuNR@SiO_2@MnO_2 (ASM), 12 mL of $KMnO_4$ (1.5 mg/mL) was dropped into the 0.4 mg/mL of AS solution under ultrasonic bath at 4 °C for 1 h.[20b] The mixture was purified by centrifugation (5000 rpm, 10 min) for three times to get the ASM solution. The Mn^{2+} concentration of ASM was measured by ICP-AES after dissolution with different buffers (The content of Mn is 14%).

The DNA prodrugs of H1 to H3 were prepared by respectively incubating H1a (200 nM) and H1b (200 nM), H2a (200 nM) and siRNA (200 nM), H3a (200 nM) and ASO (200 nM), H4 (200 nM) at 37°C for 1 h.

To prepare AuNR@SiO_2@MnO_2@DNA prodrug (ASMD), the ASM solution (100 μg/mL) were

dispersed in the DNA prodrugs (aq) (containing 200 nM of H1, H2, H3, and H4) (Table S1) and kept stirring at room temperature for 30 min. The obtained ASMD were collected by centrifugation (5000 rpm, 10 min) and dispersed in PBS buffer. The AuNR@SiO_2@DNA prodrug (ASD) was obtained by using the AS instead of ASM with the same procedure. The ASM-Cy5, ASD-FAM/TAMARA, ASMD-FAM/TAMRA, ASMD' without fluorescence/quencher pair were also prepared with the same procedure by replacing the DNA prodrugs with Cy5 labeled DNA, H2/H3 with H2-FAM/H3-TAMRA or H2'/H3' (Table S1), respectively.

2. In vitro infrared thermal imaging of ASMD

400 μg/mL of ASMD were injected in Eppendorf tubes and exposed to 1064 nm laser under the power density of 0.8 W/cm^{-2} for 5 min. The real-time temperature of the solutions was recorded with the IR imaging instrument (Fotric 225-1, China). The photothermal stability of ASMD was examined by five cycles of irradiation (1064 nm laser, 0.8 W cm^{-2}) for 5 min followed with cooling down for another 5 min to record the temperature curve.

3. In vitro degradation of ASMD

Different concentrations of ASMD solutions were dispersed in different PBS buffer systems (pH 7.4, pH 6.0, and pH6.0 containing 1 mM of GSH) at room temperature for 20 min. The obtained solutions were directly subjected to T_1 MR imaging and longitudinal relaxivity analysis. The contents of DNA prodrug and Mn^{2+} were measured by the UV-Vis spectrometry, gel electrophoresis and ICP-AES analysis.

4. Fluorescence spectrometry assay of the cascade response of DNA prodrugs

The mixture of DNA prodrugs (containing 200 nM of H1, H2, H3 and H4) incubated with different combinations of miRNA-21 (40 nM) and Mn^{2+} (1 mM) at 37 °C for 2 h. The fluorescence spectra of the mixtures ranging from 500 nm to 600 nm and 560 nm to 660 nm were recorded with excitations at 480 nm and 540 nm with a slit width of 10 nm, respectively.

5. Native polyacrylamide gel electrophoresis (PAGE) assay of the DNA prodrugs

The activity of the DNAzyme in H1 were investigated by incubating H1 with different concentrations of Mn^{2+} at 37 °C for 2 h. The Mn^{2+} and miRNA-21 synergistically initiated cascade response of DNA prodrugs was investigated by incubating different combinations of H1 to H4(1 μM) with miRNA-21(1 μM) and Mn^{2+} (1 mM) at 37 °C for 2 h.

Each product (4 μL) was mixed with 1 μL of 6 × loading buffer and 1 μL of 6 × UltraPower dye and loaded into a polyacrylamide gel (8%), which was run at 110 V in 1×TBE buffer (90 mM Tris, 89 mM boric acid and 2.0 mM EDTA, pH 8.0) for 40 min at room temperature. The gel was imaged with a Molecular Imager Gel Doc XR.

6. Electron spin resonance (ESR) measurements of •OH

The ASMD solution (40 μg/mL) was incubated with 100 μM of H_2O_2, 1 mM of GSH and 100 mM 5, 5-dimethyl-1-pyrroline-N-oxide (DMPO) in the PBS buffer (pH 6.0 containing 25 mM $NaHCO_3$)

at room temperature. After incubation for 1 min, the mixture was subjected to ESR measurement under 1 G field modulation, 100 G scan range, and 20 mW microwave power.

7. Cell culture

The A549 cells were cultured in F-12k complemented with 10% FBS, streptomycin (100 mg/mL) and penicillin (100 mg/mL) at 37 °C in a humidified atmosphere containing 5% CO_2. The cells were counted by Countess II automated cell counter (Thermo Fisher Scientific).

8. Cell cytotoxicity assay

A549 cells were seeded in a 96-well plate in advance. When the confluence reached ~80%, the cells were incubated with different concentrations of AuNR, AS, ASM at 37 °C for 4 or 24 h, respectively. After washed twice with PBS and incubated with fresh F-12K containing 10% CCK-8 for another 2 h, the cell plate was subjected to a Multiskan FC microplate reader with the absorbance at 450 nm.

For the investigation of the PTT/CDT/GT mediated cell-killing, the A549, MCF-7 or Hela cells were incubated with ASM or ASMD (40 μg mL^{-1}) at 37 °C for 48 h, which were further irradiated with 1064 nm laser (0.8 W cm^{-2}) for 10 min. Then, the cells were subjected to CCK-8 assay with the same procedure.

9. Cell viability and apoptosis assay

A549, MCF-7 or Hela cells seeded in 12-well plates (~2 × 10^5 cells/well) were incubated with 2 mL ASM or ASMD solutions (40 μg/mL in fresh culturing medium) for 48 h, part of which were irradiated with 1064 nm laser (0.8 W/cm^2) for 10 min. Then the cells were subjected to living/dead cell staining with propidium iodide (PI)/calcine AM according to the user manual and CLSM imaging. After digested from the dishes and washed with PBS, the cells were subjected to living/apoptosis staining with AnnexinV–FITC and PI according to the user manual or flow cytometer assay.

10. CLSM imaging of cells

For the investigation of the cellular uptake efficiency of ASMD, A549 cells seeded in confocal dishes (~1 x 10^4 cells/dish) were incubated with 150 μL of ASMD solution (40 μg/mL in PBS) at 37 °C for different time. After washed with PBS for three times to remove excess ASMD, the cells were subjected to CLSM imaging with a 63x oil objective, and the Cy5 channel was collected from the emission signal between 650 to 750 nm under a 633 nm excitation.

For the investigation of the cellular uptake pathway of ASMD, A549 cells seeded in confocal dishes (~1 x 10^4 cells/dish) were pre-treated with CPZ (50 μM), NYS (100 μM), and EIPA (100 μM) at 37 °C for 30 min, which were then incubated with ASMD and subjected to CLSM imaging following the same procedure.

For the investigation of the ROS generated inside cells, A549 cells seeded in confocal dishes (~1 x 10^4 cells/dish) were incubated with 150 μL of different concentrations of ASMD solution at 37 °C for 6 h. After washed with PBS for three times to remove excess ASMD, the cells were

incubated with DCFH-DA (10 μM) at 37 °C for 30 min. The obtained cells were washed with PBS and subjected to CLSM imaging.

For the investigation of the Mn^{2+} and intracellular miRNA-21 synergistically triggered release of DNA prodrugs, A549 cells seeded in confocal dishes (~70% confluence) were incubated with Lipo2000 coated anti-microRNA21 at 37 °C for 4 h according to the user manual. After washed by PBS, the cells were treated with ASD (40 μg/mL) and ASMD' (40 μg/mL) at 37 °C for 6 h, respectively. After another washing by PBS, the cells were stained with Hoechst 33342 at room temperature for 10 min and subjected to CLSM imaging with a 63x oil objective. The Hoechst 33342, FAM and TAMRA channel were respectively collected from the emission signal between 440 to 470 nm under a 405 nm excitation, 500 to 530 nm under a 488 nm excitation, and 560 to 600 nm under a 540 nm excitation.

For the investigation of the possible lysosomal trapping, A549 cells seeded in confocal dishes (~1 x 10^4 cells/dish) were incubated with 150 μL of ASMD solution (40 μg/mL) at 37 °C for 6 h. After washed with PBS, the cells were stained with Lyso-Tracker green at 37 °C for 1 h according to the user manual and subjected to CLSM imaging with a 63x oil objective, and the Cy5 channel was collected from the emission signal between 650 to 750 nm under a 633 nm excitation.

All images were digitized and analyzed with Leica Application Suite Advanced Fluorescence (LAS-AF) software package.

11. Western blotting and qRT-PCR analysis of PLK1 expression

A549 cells seeded in 6-well plates (~4 × 10^5 cells/well) were divided into four groups (G1-G4). G1 was incubate in 1.5 mL F-12K medium without further treatments. G2 was pre-treated with Lipo2000 coated anti-miRNA-21 and then incubated with ASMD 37 °C for 48 h in 1.5 mL F-12K medium. G3 and G4 were respectively treated with ASD and ASMD at 37 °C for 48 h in 1.5 mL F-12K medium.

For the western blotting analysis of PLK1 protein, the cells of different groups were treated with RIPA lysis buffer to extract the total protein, which was quantified by a BCA protein quantification kit and then separated with SDS-PAGE. The proteins inside gel were transferred to a polyvinylidene fluoride (PVDF) membrane by a wet transfer cell (Bio-rad). The obtained PVDF membrane was blocked by 5% BSA in 1×PBST buffer at room temperature for 1 h. After washing three times with 1×TBST buffer, the PVDF membrane was incubated with anti-PLK1 or anti-GAPDH mouse monoclonal antibody (1:1000 dilution) at 4 °C overnight. The resulting PVDF membrane was washed three times with TBST solution and incubated with HRP-labeled secondary antibody (1:5000 dilution) in 5% BSA-PBST for at room temperature 1 h. Finally, the membrane was washed three times with TBST solution, and covered with the ECL detection reagent and imaged by PowerPac™ basic electrophoresis analyser according to the user manual.

For qRT-PCR analysis of PLK1 mRNA, the cells of different groups were treated with FastPure Complex Cell/Tissue Total RNA Isolation kit to isolate the total RNA. The cDNA was

obtained from each group through PrimeScript RT reagent Kit with gDNA Eraser. Obtained cDNA was used as a template for qPCR amplification with PCR primers (Table S1) according to the protocol of TB Green® Premix Ex Taq™ II (Tli RNaseH Plus). The transcription of target gene was normalized to the GAPDH.

12. Hemolysis Test of ASMD

Pure erythrocytes were obtained from whole mouse blood by centrifugation (3500 rpm, 15 min) for 5 times to remove the serum and then dispersed in 5 times the volume of blood in isotonic PBS. 50 μL erythrocytes solutions were respectively added with 150 μL ultrapure water (positive), PBS (negative), ASMD (20, 40 or 80 μg mL^{-1}). The mixtures were incubated in 37 °C for 48 h and centrifuged at 3500 rpm for 15 min. The hemolysis rate was calculated using the absorbances (*A*) at 570 nm as follows:

$$\text{Hemolysis rate}(\%) = \frac{A_{\text{test}} - A_{\text{negative}}}{A_{\text{positive}} - A_{\text{negetive}}}$$

13. Animal experiments

The animal experiments were performed according to the Laboratory Animal Management of Jiangsu Province for the care and use of laboratory animals (License No. SYXK 2019-0056) approved by the Department of Science and Technology of Jiangsu Province (Nanjing, China) and the Institutional Animal Care and Use Committee (IACUC) of Nanjing University. BALB/c nude mice (female, 4 weeks) were purchased from Jiangsu Cavens Laboratory Animal Co., Ltd. (China). To establish the A549 subcutaneous tumor xenograft mice, A549 cells (1.0×10^7 cells) suspended in 100 μL of HBSS medium (containing 50% (v/v) matrigel) were subcutaneously inoculated into the root of right hind leg of each mouse. After the tumor volume reached 100-150 mm^3, the tumor-bearing mice were treated with different nanodrugs by peritumoral injection and subjected to PA/MR/FL imaging and PTT/CDT/GT.

For in vivo PA/MR/FL imaging, the A549 tumor-bearing mice were peritumorally injected with ASMD (dose: 4 mg/kg) and subjected to in vivo animal imaging at different time nodes. The PA imaging was performed by a Vevo LAZR-X imaging system (FUJIFILM VisualSonics, Canada). The MR imaging was performed on a high magnetic field micro-MR scanner (3.0 T, U790 United Imaging. China) by using a mouse coil. The FL imaging was acquired using an IVIS Lumina XR III Imaging System. The thermal imaging was performed by irradiating the tumor region with the 1064 nm laser (0.8 W cm^{-2}) for 5 min and monitoring the real-time temperature with an IR imaging instrument (Fotric 225-1, China). Each imaging experiment was conducted on three mice.

For in vivo PTT/CDT/GTT, the A549 tumor-bearing mice were weighted and randomly divided into six groups (5 mice per group): (1) saline, (2) saline with NIR irradiation, (3) ASM, (4) ASMD, (5) ASM with NIR irradiation, (6) ASMD with NIR irradiation. Groups (1) and (2) were peritumorally injected with 100 μL of PBS. Groups (3) and (5) were peritumorally injected with 100 μL of ASM (dose: 4 mg/kg). Groups (4) and (6) were injected with 100 μL of ASMD (dose:

4 mg/kg). After injection for 4 h, groups (2), (5) and (6) were irradiated with 1064 nm laser (0.8 W/cm^2) for 10 min. The injection and irradiation procedures were repeated every other day for 14 days. The tumor volume and body weight of each mouse were recorded with a digital vernier caliper and an electronic balance every other day. The tumor volume (*V*) was calculated as $V = (L \times W^2)/2$, while *L* and *W* represents the length and width of the tumor, respectively. At day 15, all mice were sacrificed, and the tumors, hearts, kidneys, spleens, lungs, and livers were dissected from the euthanized mice. The photo of the tumors from each groups of mice were taken. For histopathological analysis, the dissected tumors and other organs were washed with PBS and fixed in 4% paraformaldehyde solution, which were further embedded in paraffin, sectioned and stained with the H&E staining kit to acquire the images with an optical microscope. The tumor slices were also performed TUNEL assay and CLSM imaging of the DAPI and FITC channels, which was respectively collected from the emission signal between 430 and 500 nm or 505 and 575 nm under a 405 nm or 495 nm excitation.

Blood circulation was analyzed by measuring the contents of Au in the blood drawing from the mice after injection of ASMD at different time points by ICP-MS. The blood auto-Au level was measured from blank blood samples of mice without ASMD injection, which was subtracted when calculating the Au contents of blood samples from ASMD injected mice. The metabolic circulation was analyzed by measuring the contents of Au from the tissues including heart, liver, spleen, lung, kidney and tumors, which were collected at different time points and digested by HNO_3 and H_2O_2 mixture (v/v ratio is 7:1) at 70 °C until the mixed solutions became colorless and clear. The Au contents in all samples was measured by ICP-MS. All data were converted to %ID/g unit for dose normalization (%ID/g: percentage injection dose of nanoparticle per unit mass tissue).

14. Statistical Analysis

Data were presented as means ± standard deviation. For differences between two groups, data were analyzed using a Student's t test at a significance level of $p > 0.05$ (NS), $p < 0.05$ (*), $p < 0.01$ (**), $p < 0.001$ (***).

Table S1. Sequences of DNA prodrugs, RNA and qRT-PCR primers used in this work.

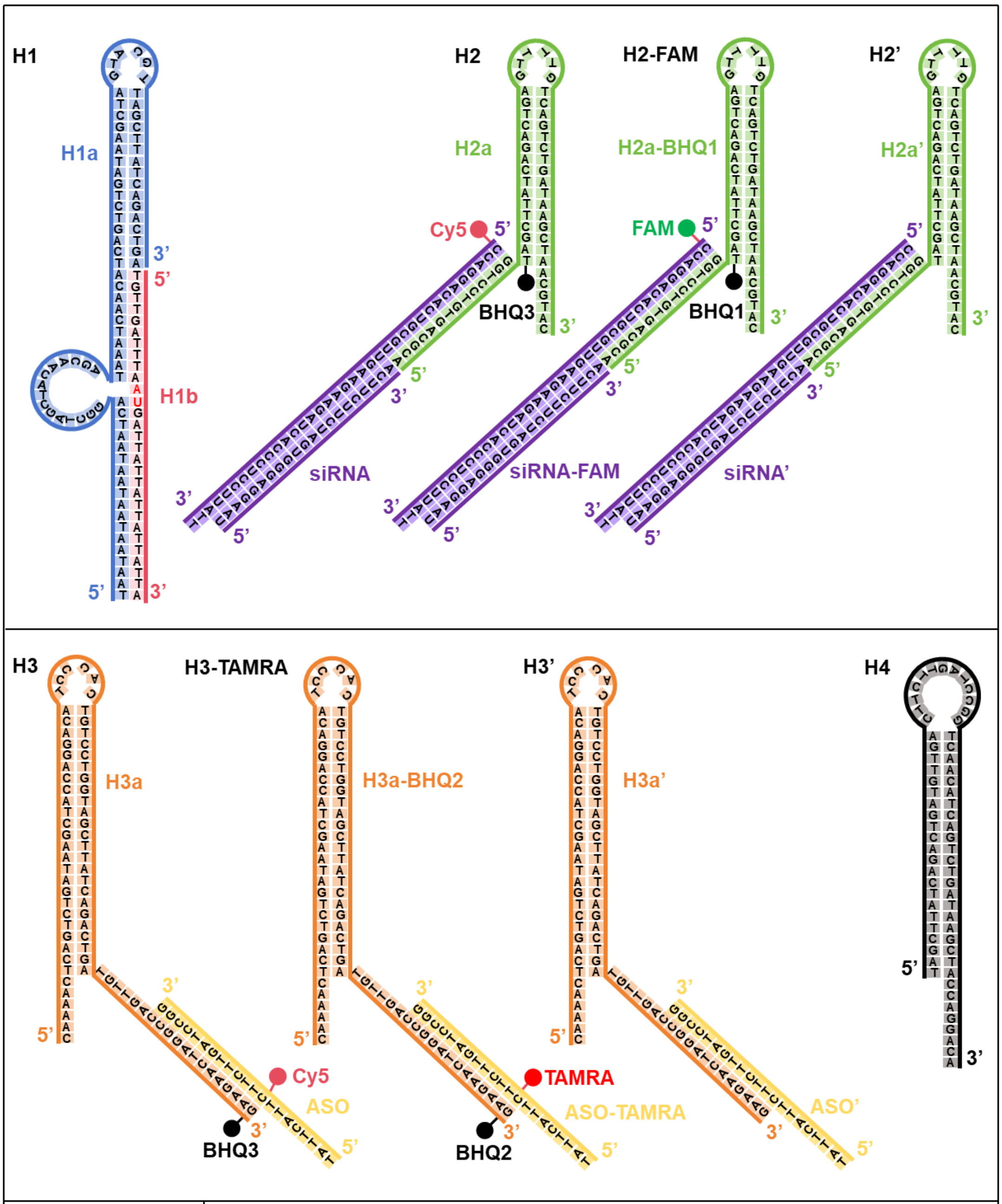

**miRNA-21**	**5'- UAGCUUAUCAGACUGAUGUUGA - 3'**
**anti-miRNA-21**	**5'- UCAACAUCAGUCUGAUAAGCUA - 3'**
**Cy5 labeled DNA**	**5'- TATTCATTC/iCy5/TTCTTGATCCGG - 3'**
**PLK1-F**	**5'- GGCAACCTTTTCCTGAATGA - 3'**
**PLK1-R**	**5'- AATGGACCACACATCCACCT - 3'**
**GAPDH-F**	**5'- CATGTTCGTCATGGGTGTGA - 3'**
**GAPDH-R**	**5'- GGTGCTAAGCAGTTGGTGGT - 3'**

Supplementary Figures

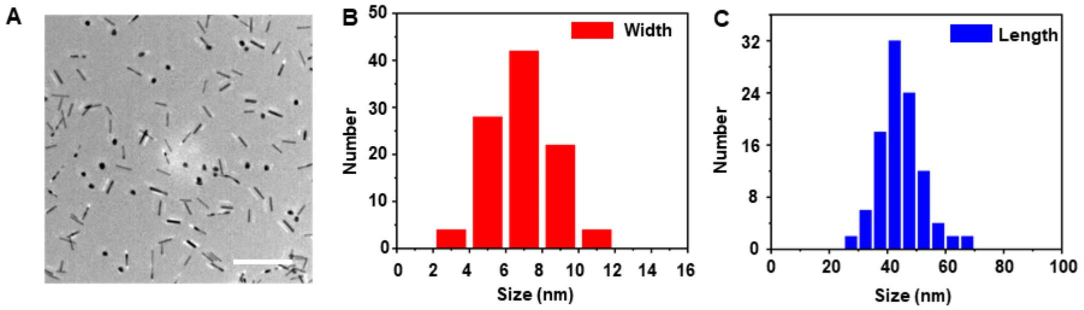

Figure S1. (A) TEM images of AuNRs. Scale bar: 200 nm. (B) Width and (C) length distributions of the of AuNRs.

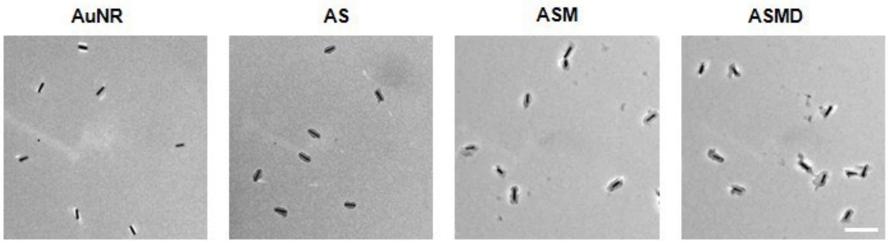

Figure S2. TEM images of AuNR, AS, ASM and ASMD. Scale bar: 200 nm.

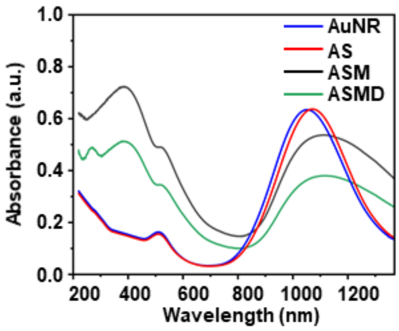

Figure S3. UV−vis-NIR spectra of AuNR, AS, ASM and ASMD.

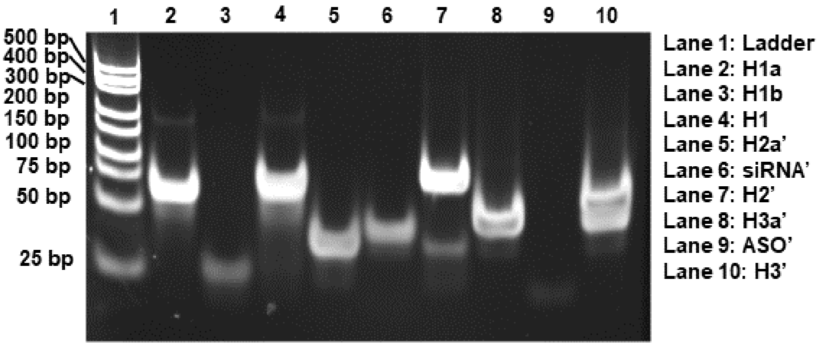

Figure S4. Gel electrophoresis analysis of the construction of the DNA prodrugs.

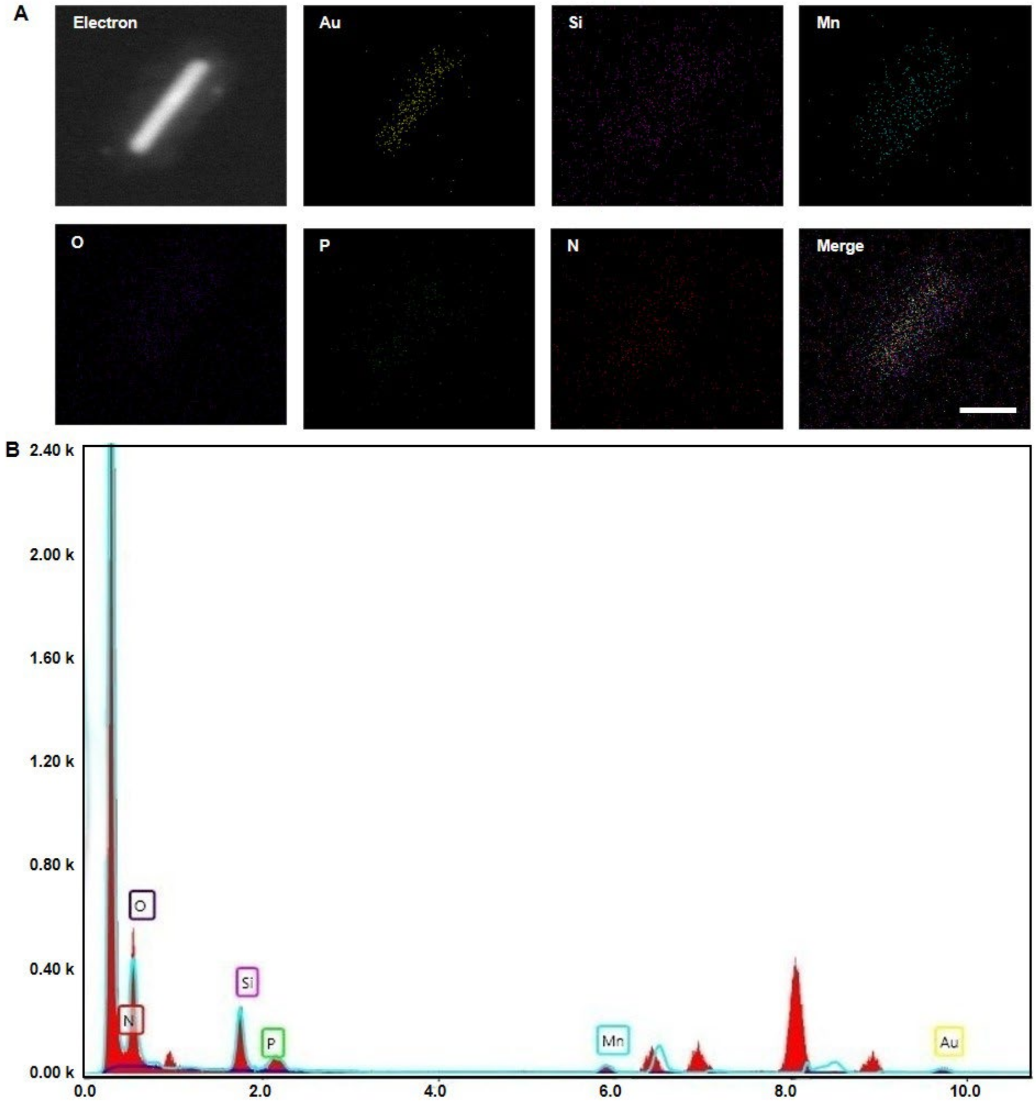

Figure S5. Element mapping images (A) and EDS spectrum (B) of ASMD.

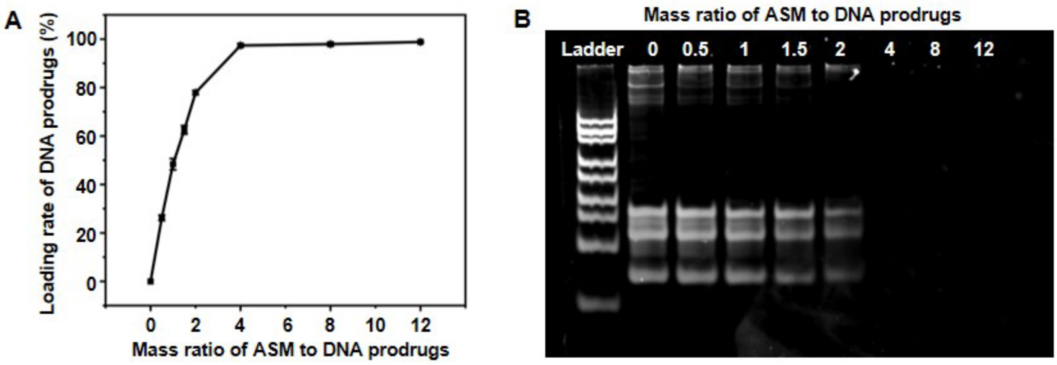

Figure S6. (A) Plots of the loading rates of DNA prodrugs vs the mass ratios of ASM to DNA prodrugs. The loading rates was calculated by subtracting the ratios of the nucleic acid characteristic 260 nm absorption peak intensities of the supernatants of DNA prodrugs after incubating with ASM and the initial solutions of DNA prodrugs from 100%. (B) Gel electrophoresis analysis of the supernatants of DNA prodrugs after incubating with ASM under different mass ratios of ASM to DNA prodrugs.

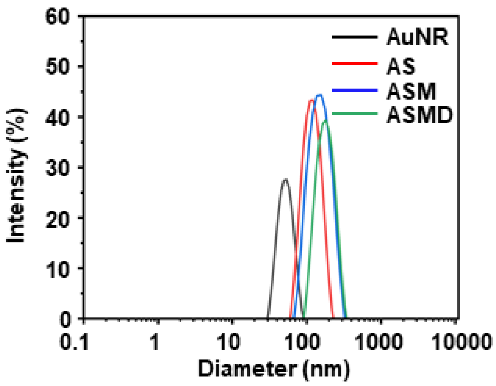

Figure S7. Hydrated diameters of AuNR, AS, ASM and ASMD.

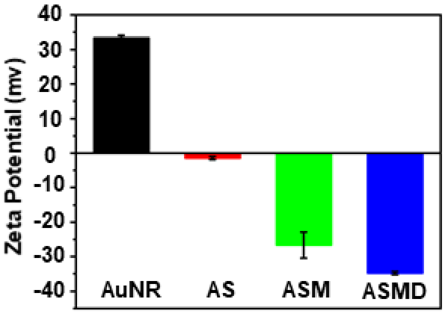

Figure S8. Zeta potentials of AuNR, AS, ASM and ASMD.

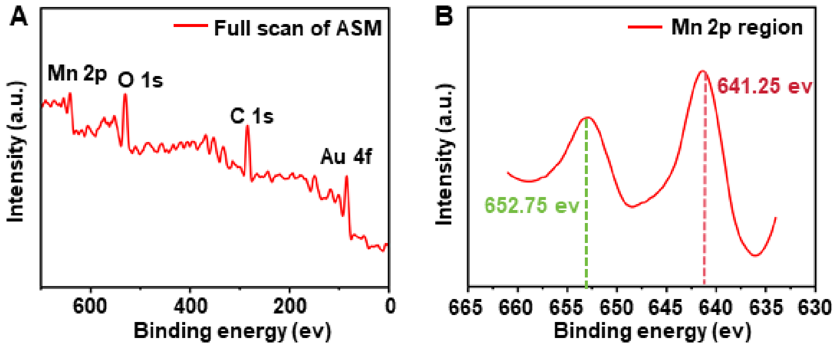

Figure S9. (A) XPS spectrum of ASM. (B) Enlarged Mn 2p region from (A).

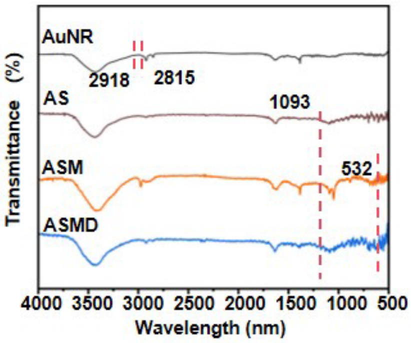

Figure S10. FTIR spectra of AuNR, AS, ASM, and ASMD.

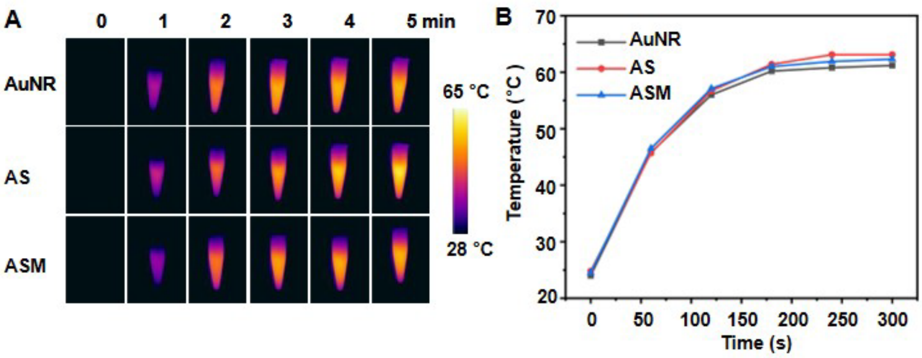

Figure S11. (A) Infrared thermal images of 400 μg/mL of AuNR, AS and ASM solution upon 1064 nm laser irradiation at a power density of 0.8 W cm^{-2} from 1-5 min. (B) Temperature curves of AuNR, AS and ASM solution from (A).

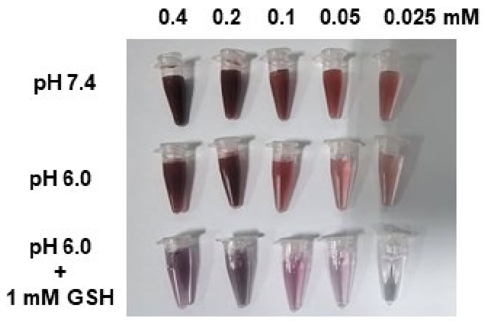

Figure S12. Photo of different Mn^{2+} concentrations of ASMD solutions under different conditions.

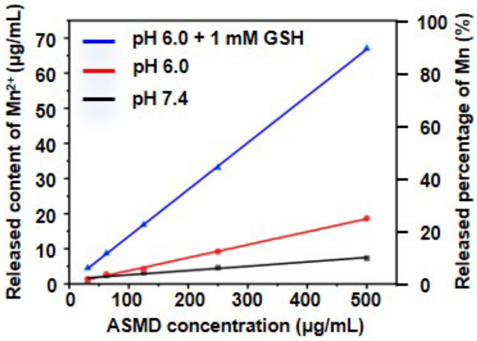

Figure S13. Plots of the released content or percentage of Mn^{2+} to ASMD concentrations at different pH or with extra GSH (1 mM).

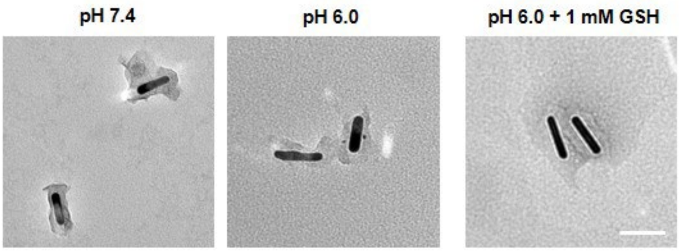

Figure S14. TEM images of ASMD under different pH or with extra GSH (1 mM) treatment. Scale bar: 50 nm.

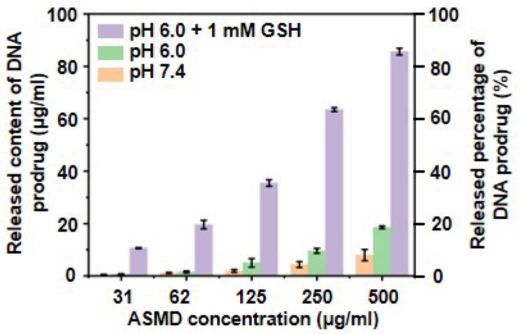

Figure S15. The released content or percentage of DNA prodrugs under different pH or with extra GSH (1 mM).

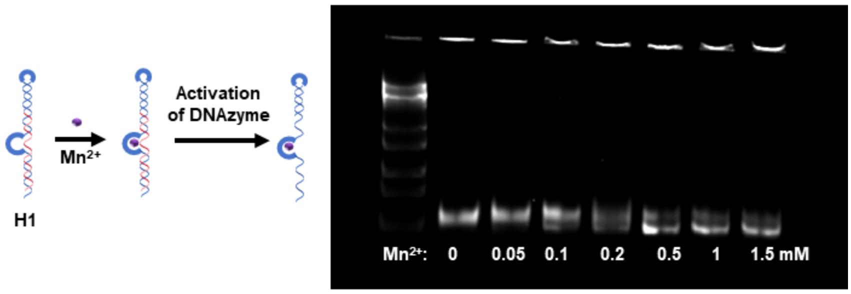

Figure S16. Gel electrophoresis analysis of H1 incubated with different concentrations of Mn^{2+}.

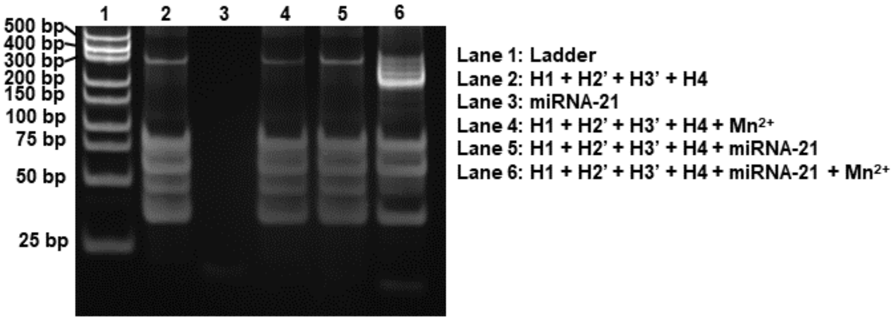

Figure S17. Gel electrophoresis analysis of Mn^{2+} and miRNA-21 synergistically initiated cascade response of DNA prodrugs.

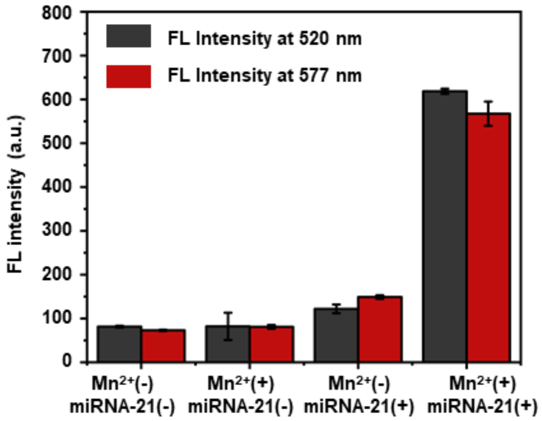

Figure S18. Fluorescence intensity at 520 or 577 nm from Figure 1H.

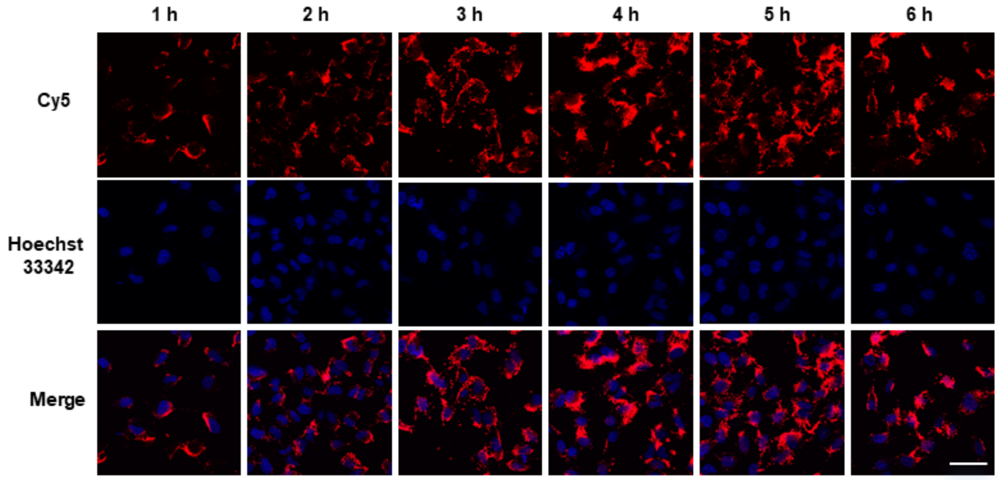

Figure S19. CLSM images of A549 cells incubated with ASM-Cy5 for different time. Scale bar: 40 μm.

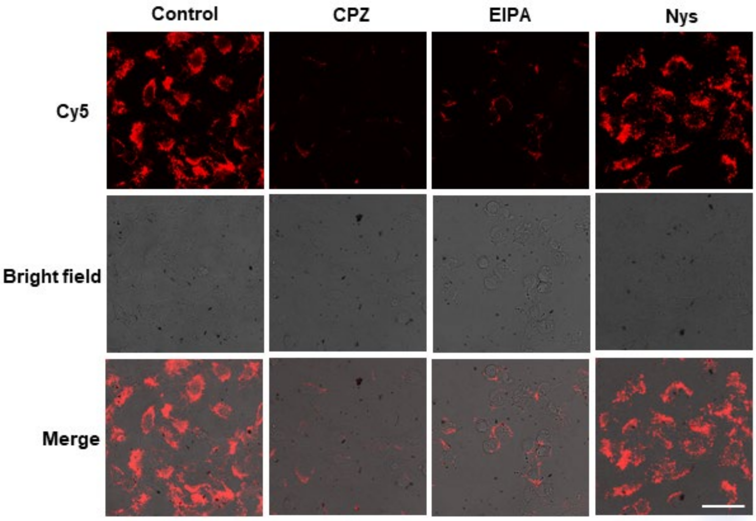

Figure S20. CLSM images of A549 cells pre-treated with CPZ, EIPA or Nys and then incubated with ASM-Cy5 for 6 h. Scale bar: 40 μm.

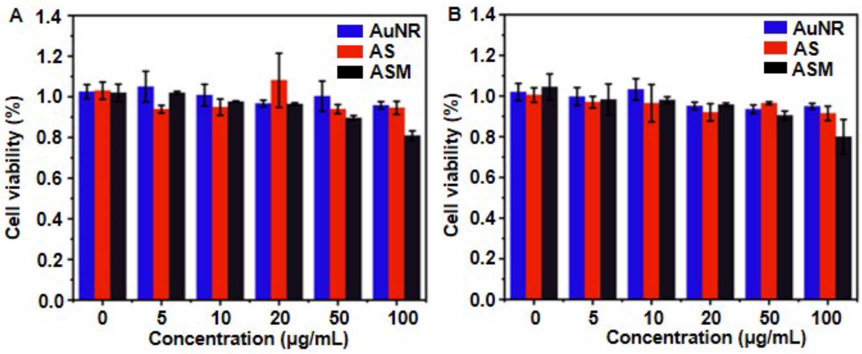

Figure S21. CCK8 assay of A549 cells treated with different concentrations of AuNR, AS and ASM for 4 h (A) or 24 h (B), respectively.

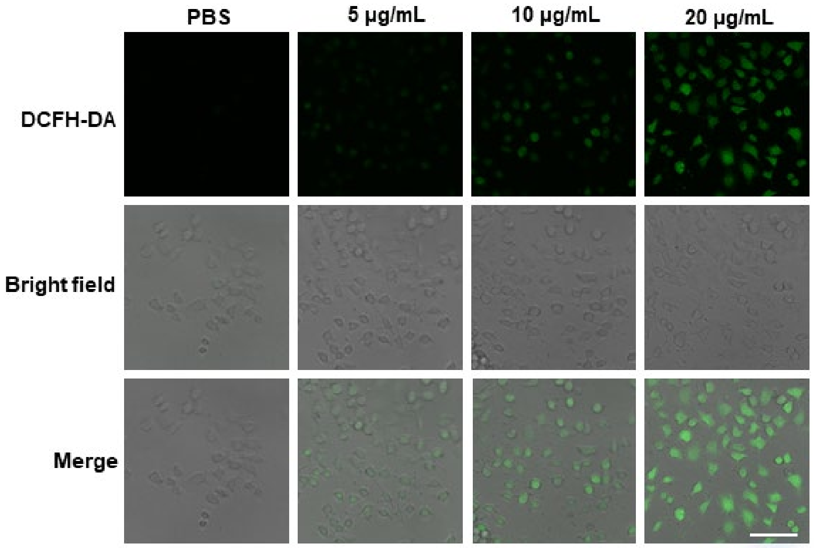

Figure S22. CLSM images of A549 cells treated with different concentration of ASM and then stained with DCFH-DA. Scale bar: 80 µm.

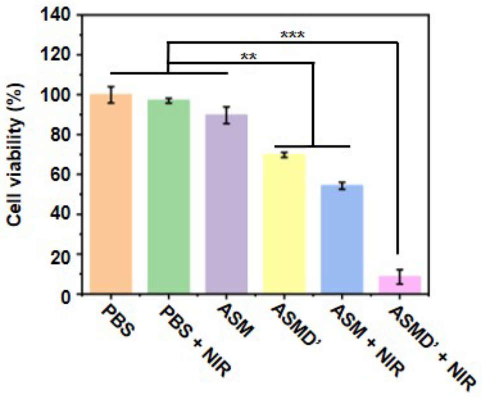

Figure S23. CCK8 assay of A549 cells treated with ASM, ASMD' with or without NIR irradiation, respectively. The data were analyzed using a Student's t test at a significance level of $p > 0.05$ (NS), $p < 0.05$ (*), $p < 0.01$ (**), $p < 0.001$ (***).

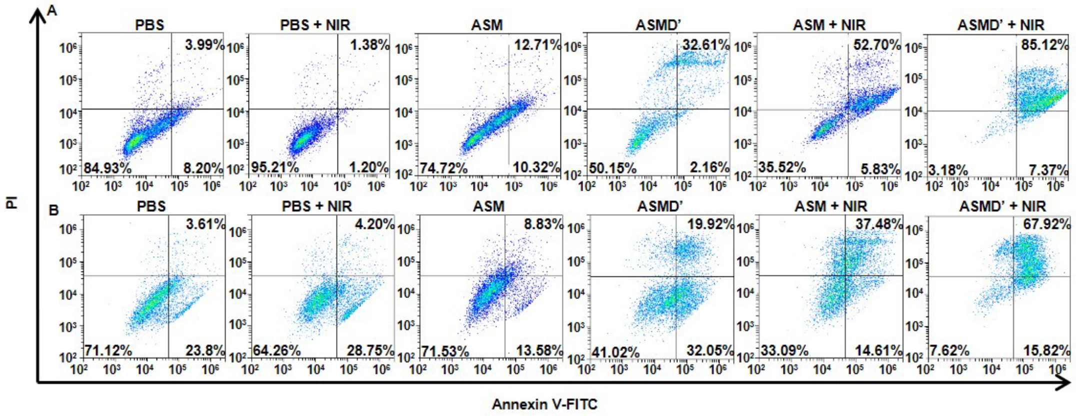

Figure S24. Flow cytometry analysis of MCF-7 (A) and HeLa (B) cells treated with PBS, ASM, ASMD' with or without NIR irradiation, respectively.

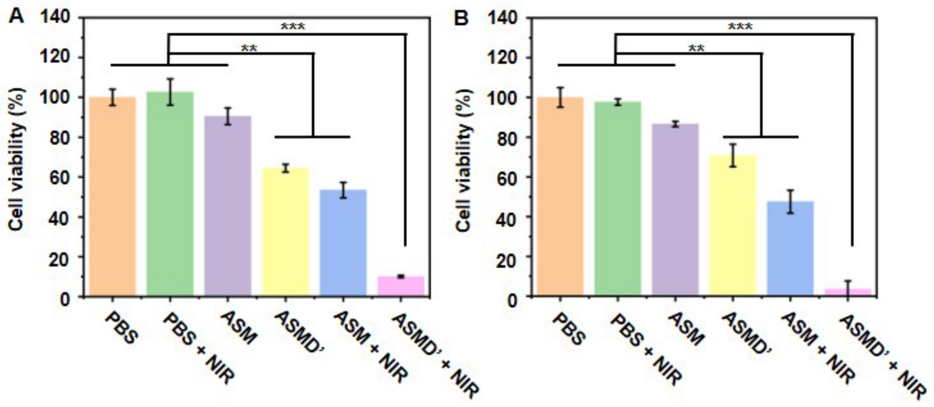

Figure S25. CCK8 assay of MCF-7 (A) and HeLa (B) cells treated with ASM, ASMD' with or without NIR irradiation, respectively. The data were analyzed using a Student's t test at a significance level of p > 0.05 (NS), p < 0.05 (*), p < 0.01 (**), p < 0.001 (***).

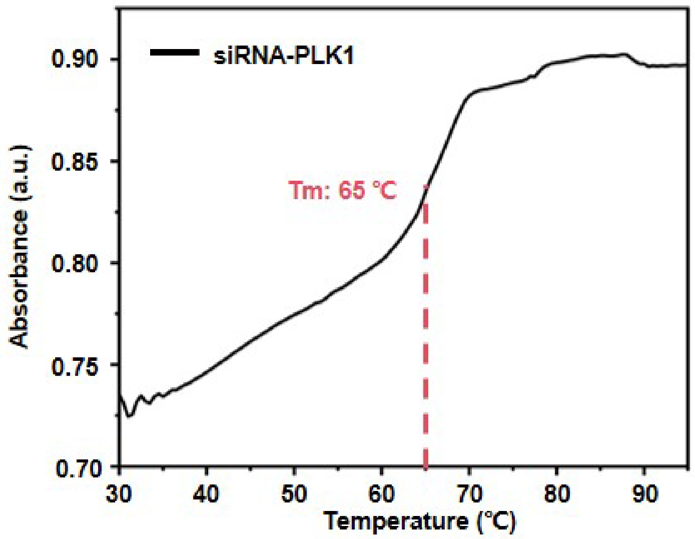

Figure S26. The melting curve of siRNA-PLK1.

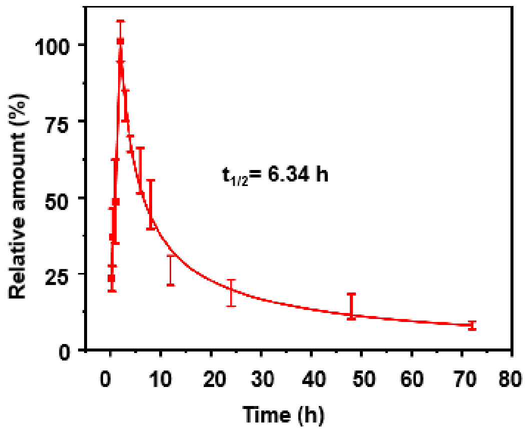

Figure S27. Blood circulation curve of the contents of Au measured by ICP-MS detection.

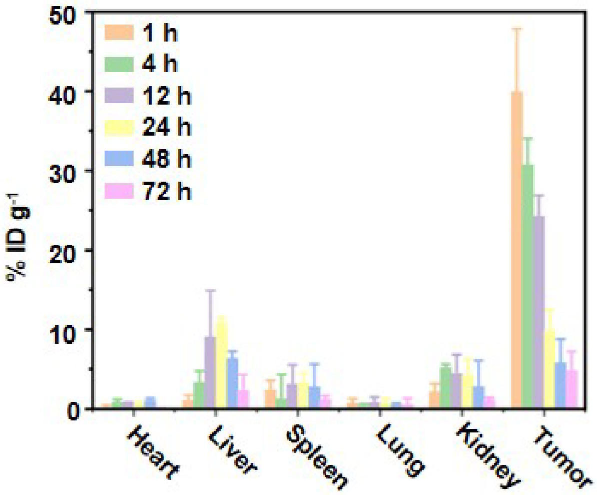

Figure S28. Metabolic circulation of the contents of Au at different post-injection time points in major organs and tumors measured by ICP-MS detection.

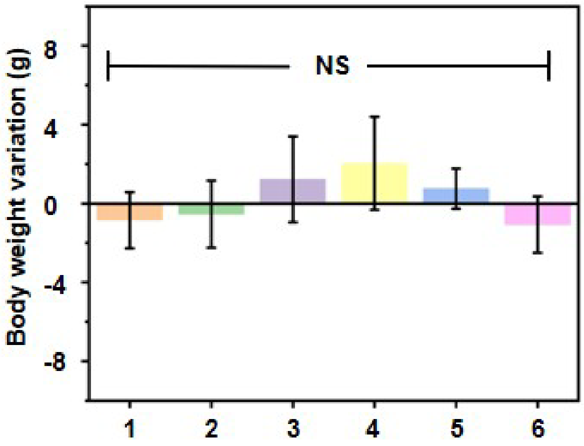

Figure S29. The body weight variations from day 0 to day 14 of the mice in different groups from Figure 4B. The data were analyzed using a Student's t test at a significance level of $p > 0.05$ (NS), $p < 0.05$ (*), $p < 0.01$ (**), $p < 0.001$ (***).

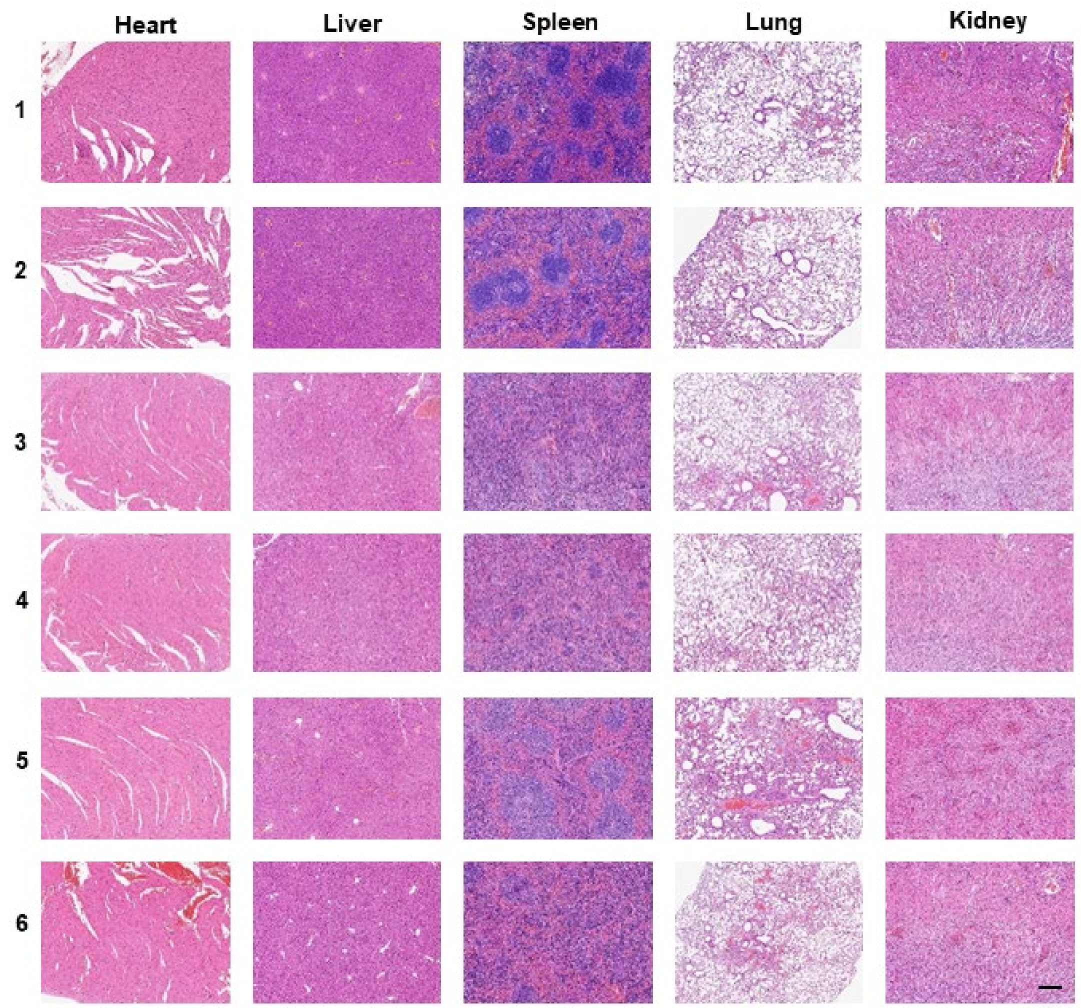

Figure S30. Histological H&E staining images of heart, liver, spleen, lung and kidney collected from different groups of A549 tumor-bearing mice. Scale bar: 400 μm.

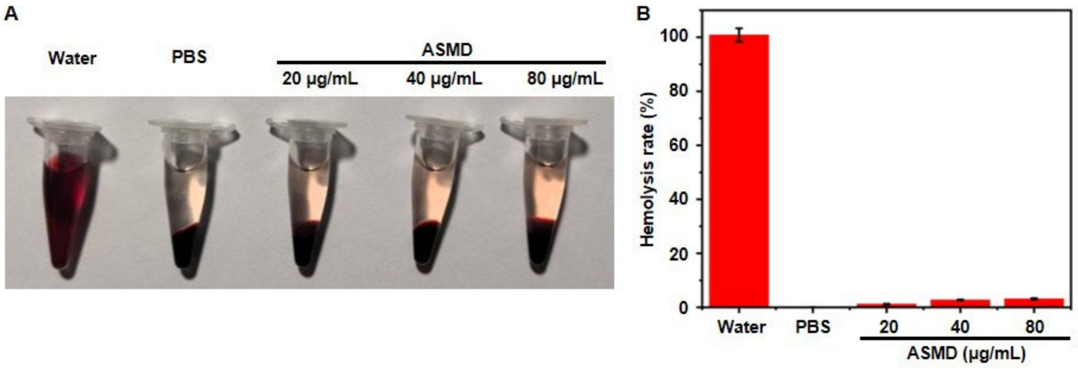

Figure S31. (A) Photographs of A549 tumor-bearing mice blood after incubated with water, PBS, and different concentrations of ASMD for 48 h. (B) Histograms of hemolysis rate from (A).

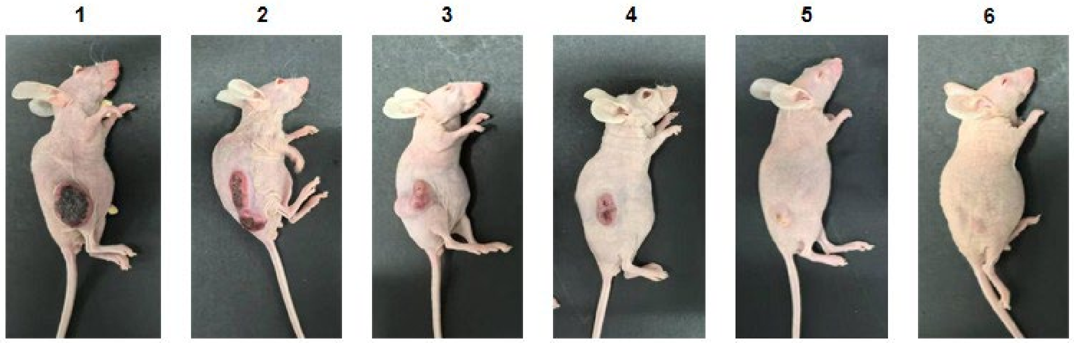

Figure S32. Representative photographs of mouse at day 14 from different groups.

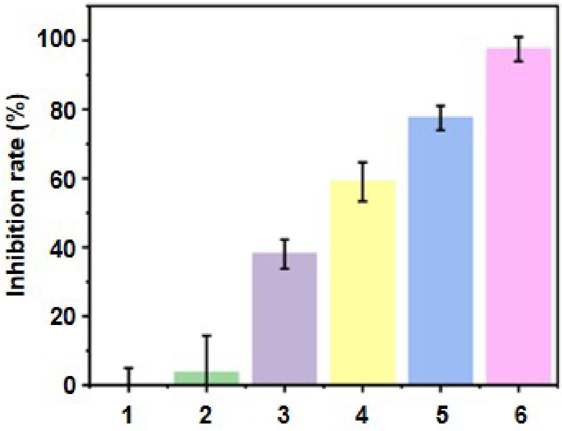

Figure S33. The tumor volume inhibition rates of mice at day 14 from different groups calculated from Figure 4C.
